# Supplementary figures and images for: Modelling the structures of frameshift-stimulatory pseudoknots from representative bat coronaviruses
Source: PLoS Comput Biol. 2023 May 19;19(5):e1011124. doi: 10.1371/journal.pcbi.1011124 (PMC10234561; doi:10.1371/journal.pcbi.1011124)

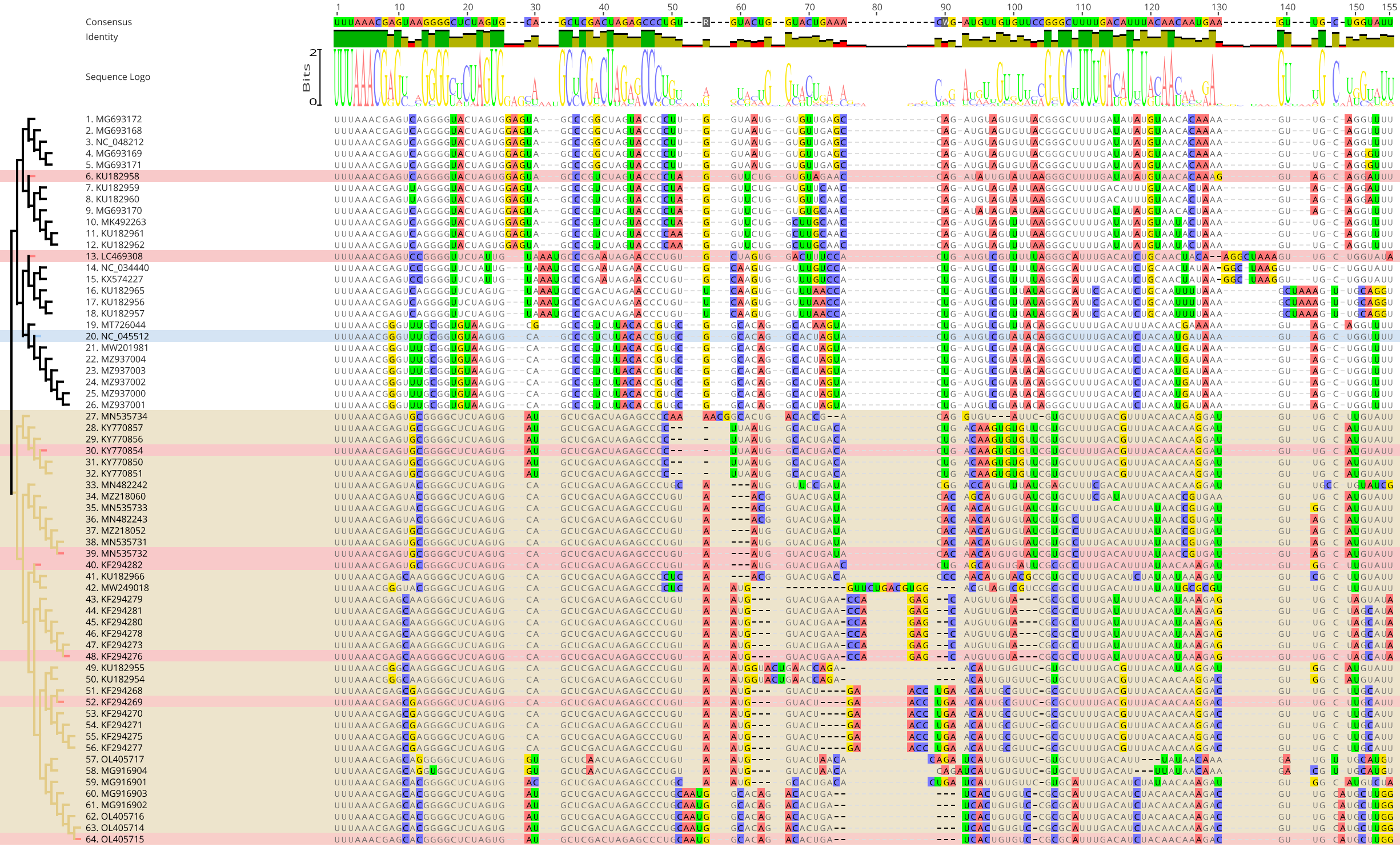

Supplement: S1 Data — PDF file containing multiple sequence alignment used for clustering bat-CoV pseudoknots. (PDF) [file pcbi.1011124.s001.pdf]

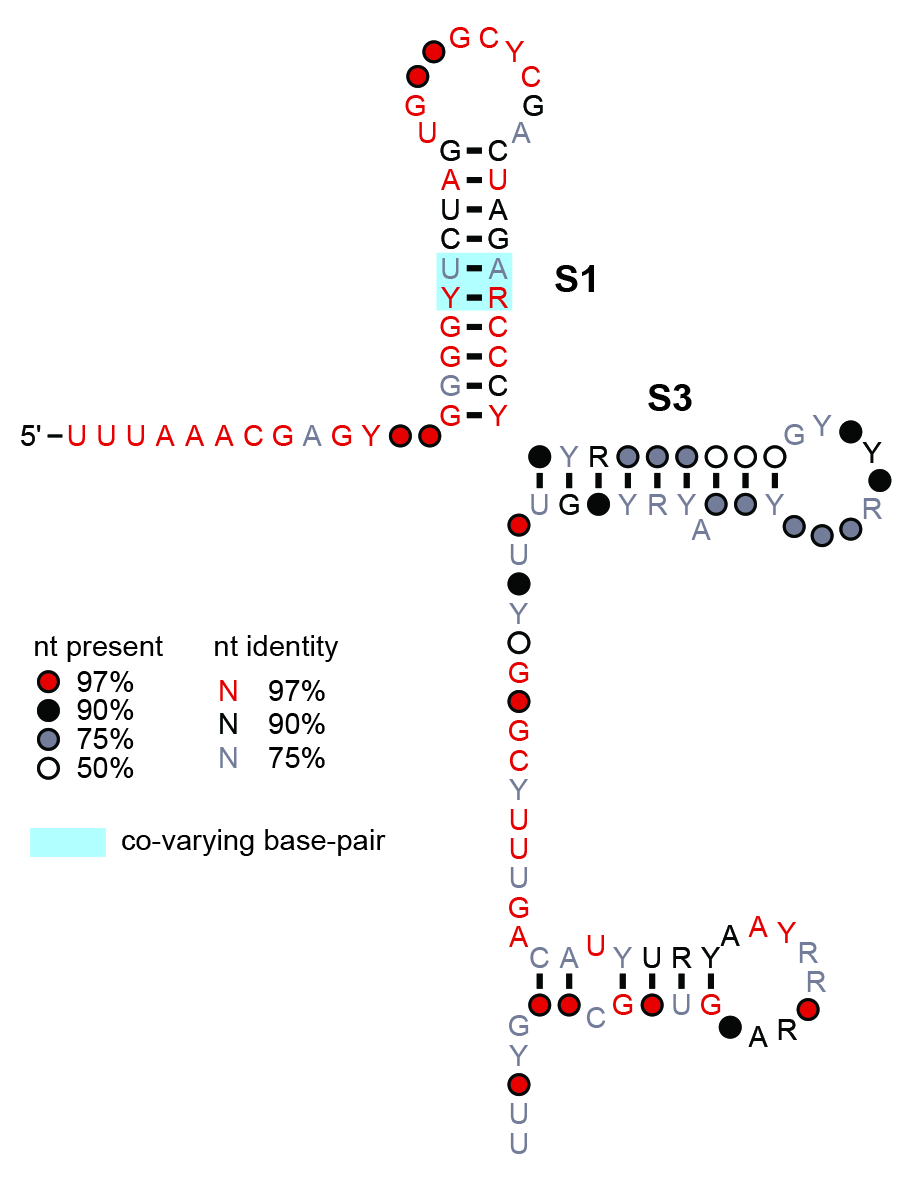

Supplement: S1 Fig — Stem 1 is highly conserved across the multiple sequence alignment, but stem 3 less so, consistent with the large variability in S3 in the predicted structures. R-scape does not evaluate stem 2 because the pseudoknotted base pairs are ignored by Infernal when constructing the covariance model. The extra stem is a hairpin downstream of the pseudoknot in the SARS-CoV-2 genome that is also less conserved. (TIF) [file pcbi.1011124.s005.tif]

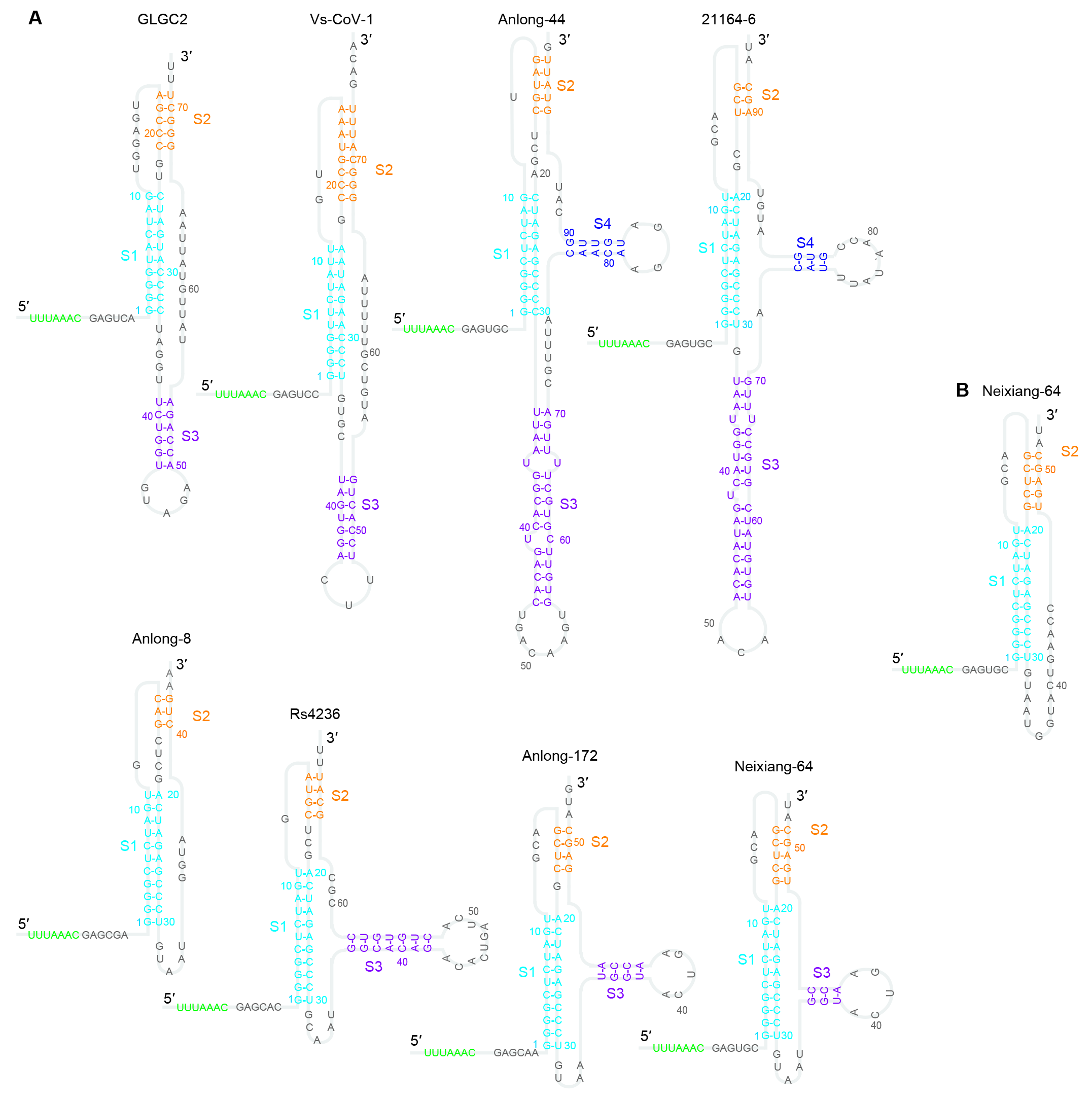

Supplement: S2 Fig — (A) Lowest-energy consensus predictions from pKiss, Hotknots, PKNOTS, and NUPACK. Cyan: S1; gold: S2; purple: S3; blue: S4; green: slippery sequence. (B) Alternative prediction for Neixiang-64 from pKiss. (TIF) [file pcbi.1011124.s006.tif]

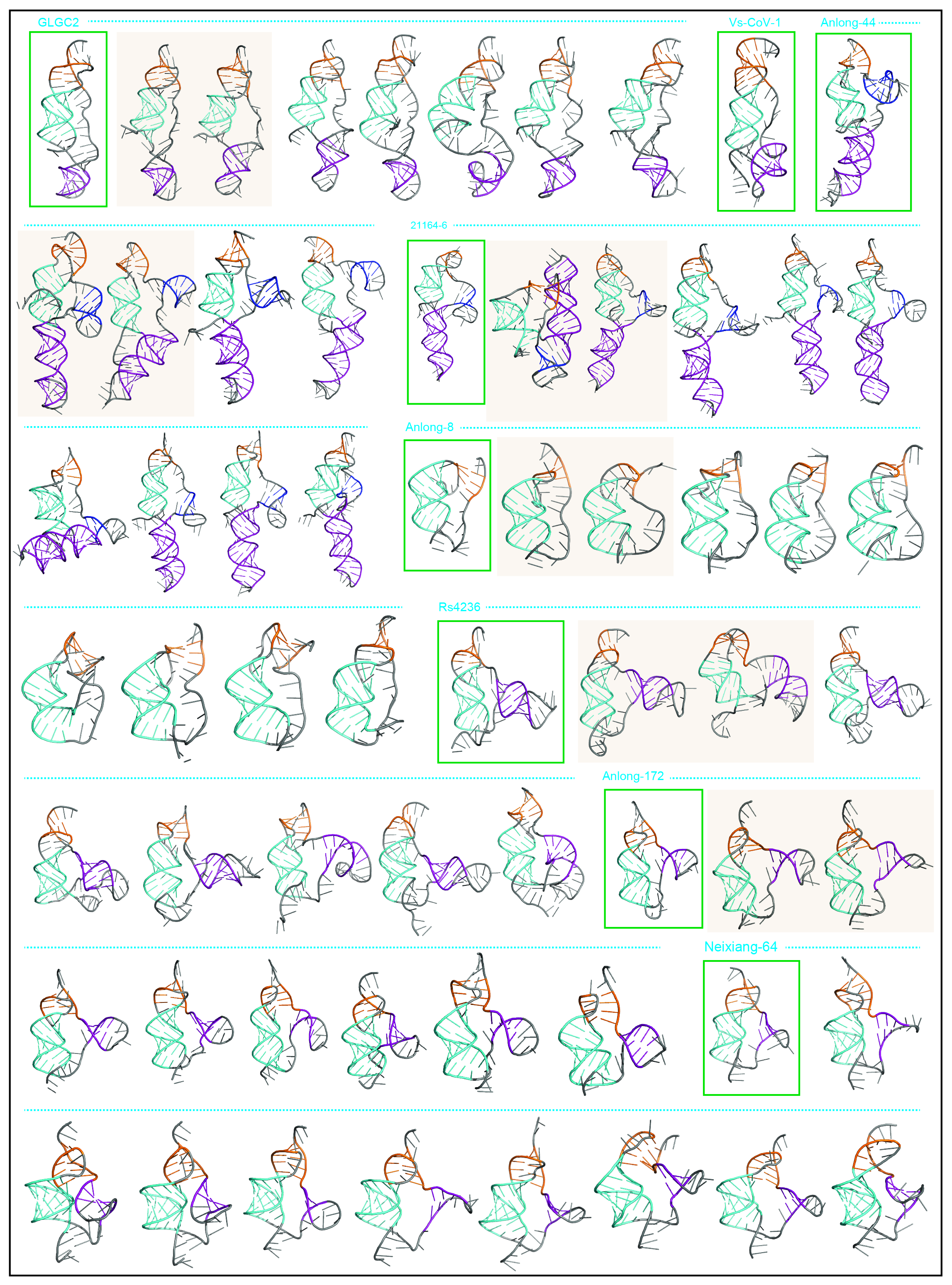

Supplement: S3 Fig — Predicted structures that featured 5′ end threading for each of the eight representative bat-CoV pseudoknots. Structures illustrated in order of increasing energy from left to right. Lowest-energy structures selected for use in MD simulations shown in green boxes. Next-lowest energy threaded conformers selected for MD replicates shaded in brown. (TIF) [file pcbi.1011124.s007.tif]

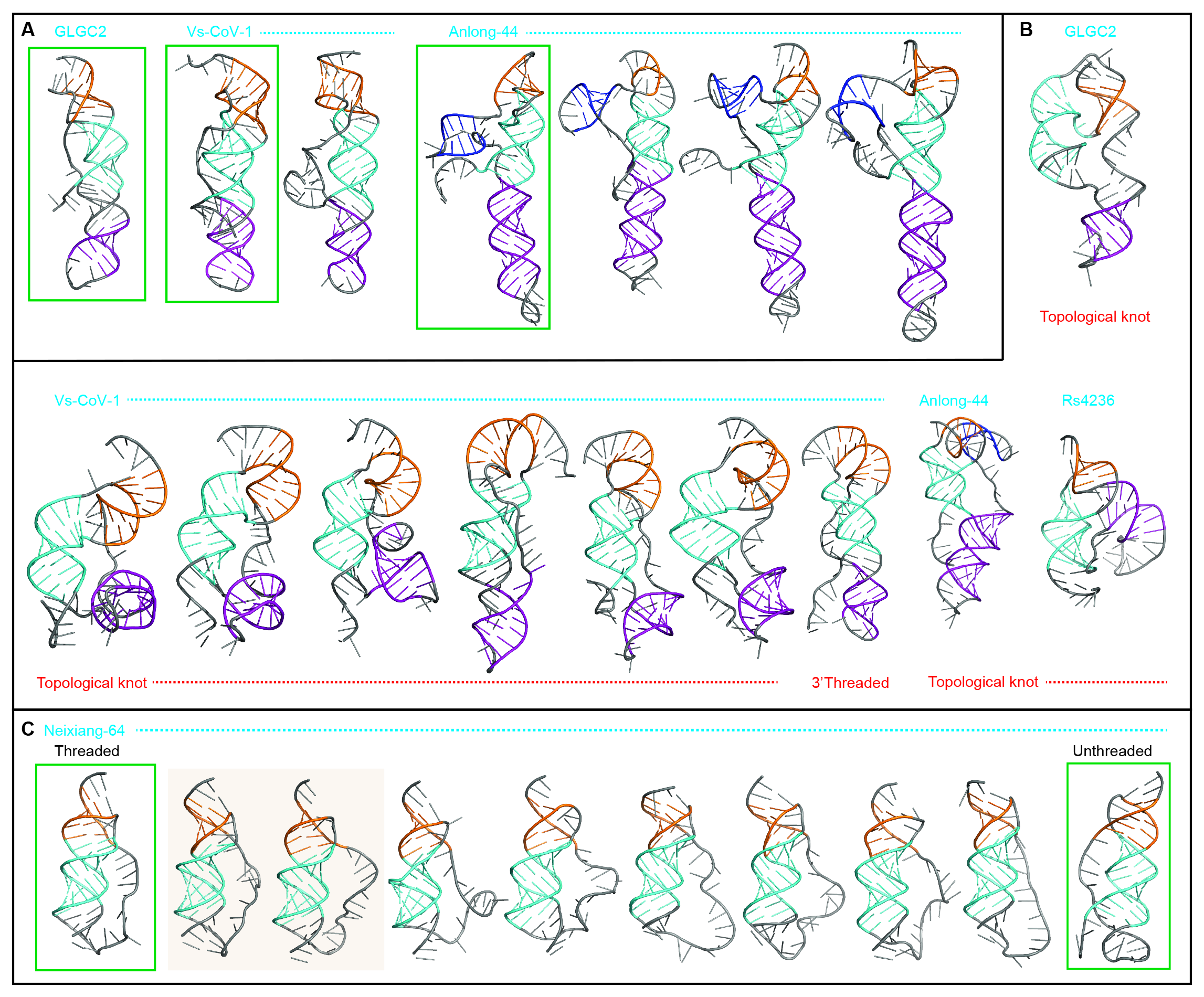

Supplement: S4 Fig — (A) Predicted structures without 5′ end threading for bat-CoV pseudoknots featuring unthreaded conformers. (B) Predicted structures rejected because they contained topological knots or 3′ end threading. (C) Alternative predictions for Neixiang-64 featuring 2-stem architecture. Structures illustrated in order of increasing energy from left to right. Lowest-energy structures for each conformer selected for use in MD simulations shown in green boxes. Next-lowest energy threaded conformers selected for MD replicates shaded in brown. (TIF) [file pcbi.1011124.s008.tif]

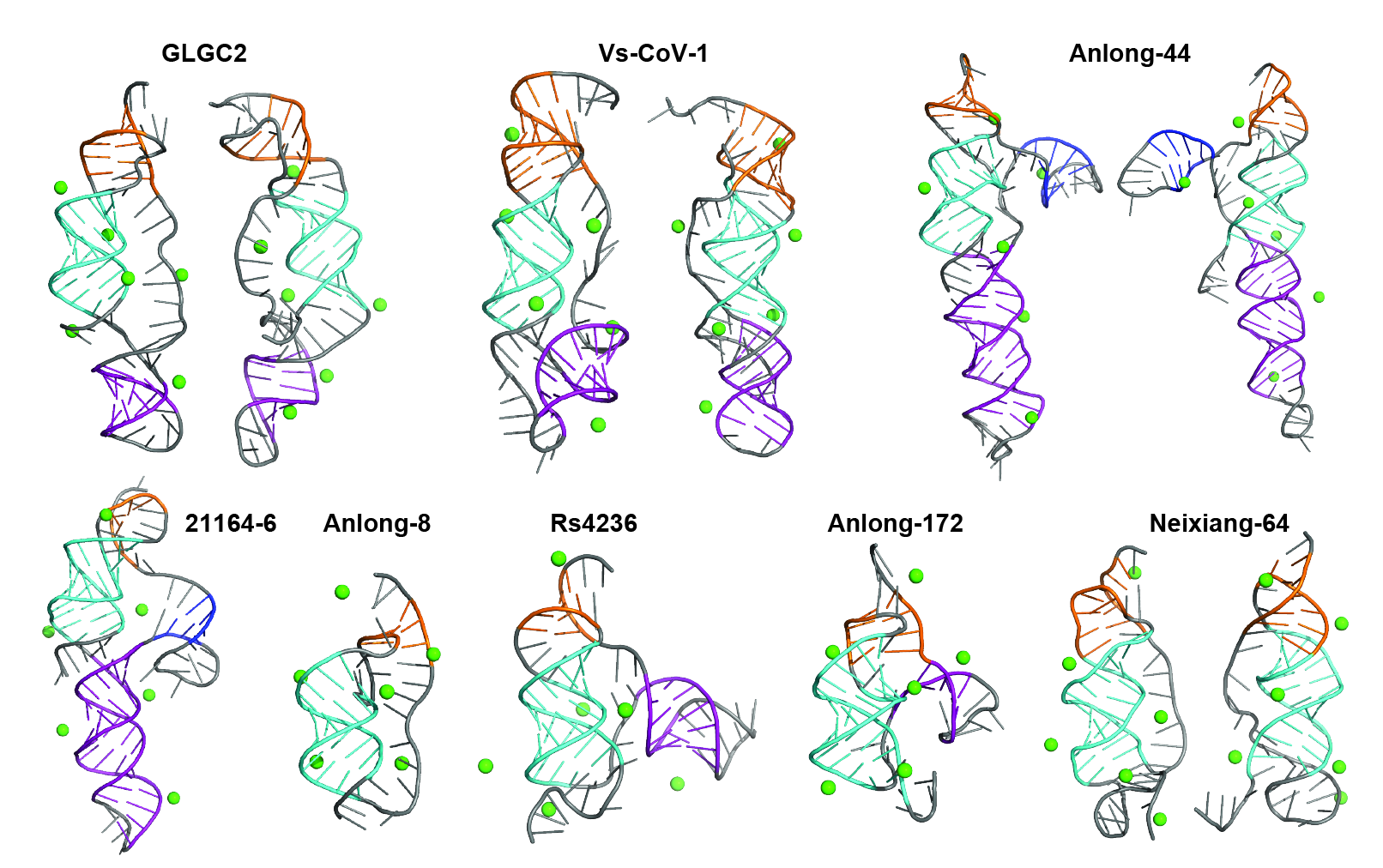

Supplement: S5 Fig — Mg2+ ions positioned by hand using MOE. (TIF) [file pcbi.1011124.s009.tif]

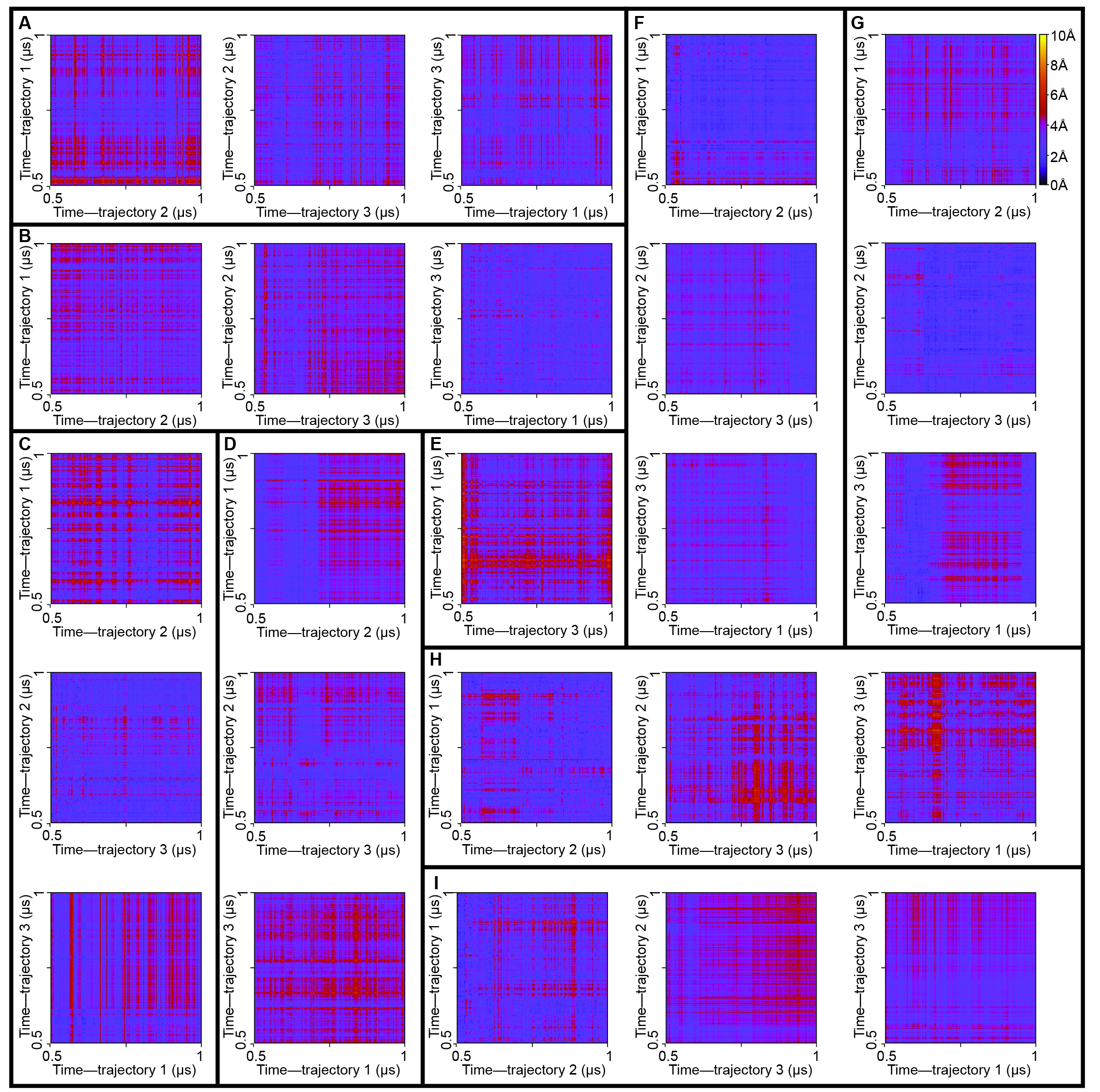

Supplement: S6 Fig — Pairwise RMSD for the structured regions of replicate trajectories for the (A) GLGC2, (B) Vs-CoV-1, (C) Anlong-44, (D) 21164–6, (E) Anlong-8, (F) Rs4236, (G) Anlong-172, and (H) Neixiang-64 pseudoknots. (TIF) [file pcbi.1011124.s010.tif]

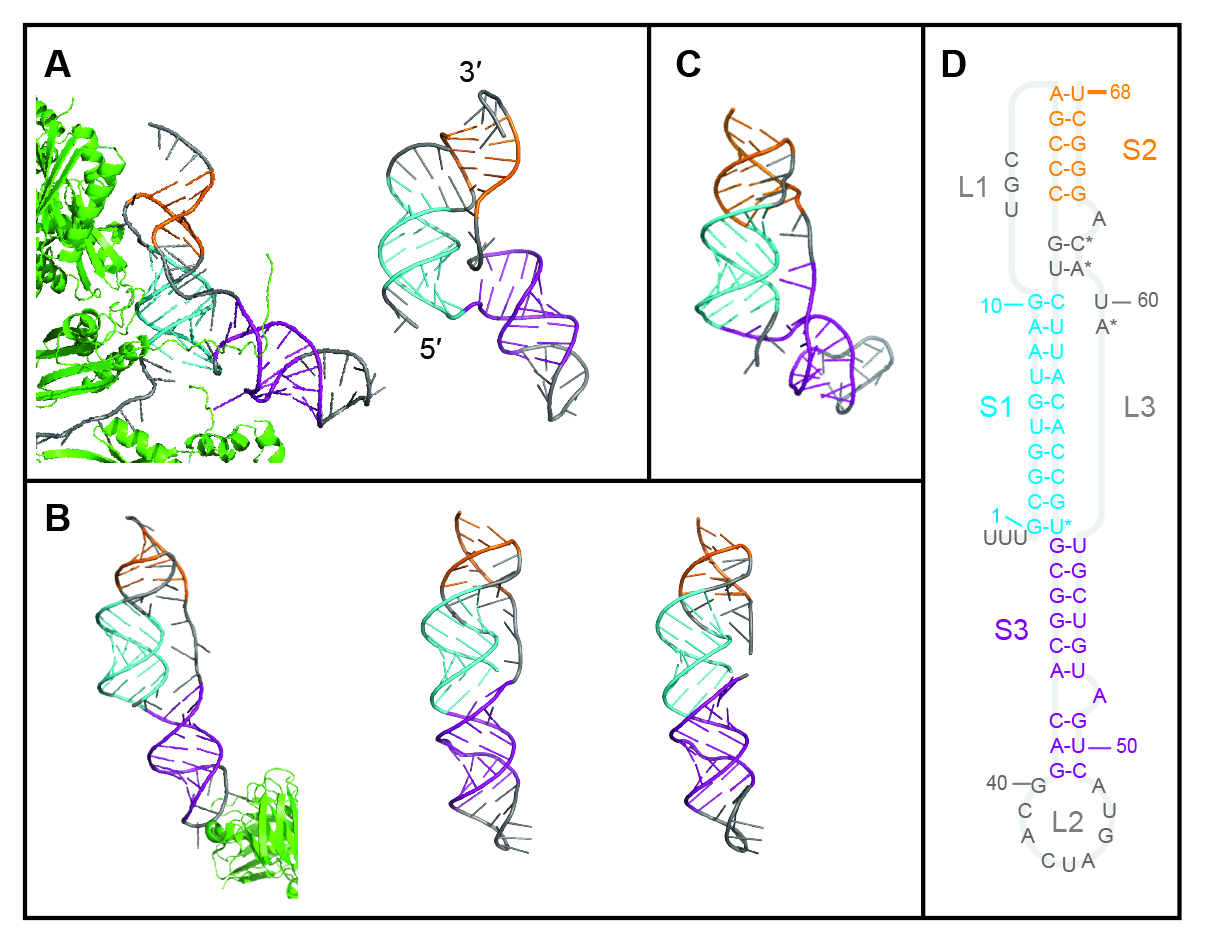

Supplement: S7 Fig — (A) Structures obtained from cryo-EM images of the pseudoknot on the ribosome (left, PDB ID: 7O7Z) and in isolation (right, PDB ID: 6XRZ). (B) Structures obtained from x-ray crystallography, complexed with a chaperone (left, PDB ID: 7MLX) and in isolation (middle, PDB ID: 7LYJ; right, PDB ID: 7MKY). (C) Threaded conformer from MD simulation of lowest-energy prediction by FARFAR2 [20]. (D) Experimentally observed base-pairing [18,21–23,48]. Asterisked base-pairs are not consistent between structures. Note that the computational model, obtained from blind 3D structure prediction as in the procedure used for the bat-CoV pseudoknots, is qualitatively similar to the experimental structures. (TIF) [file pcbi.1011124.s011.tif]

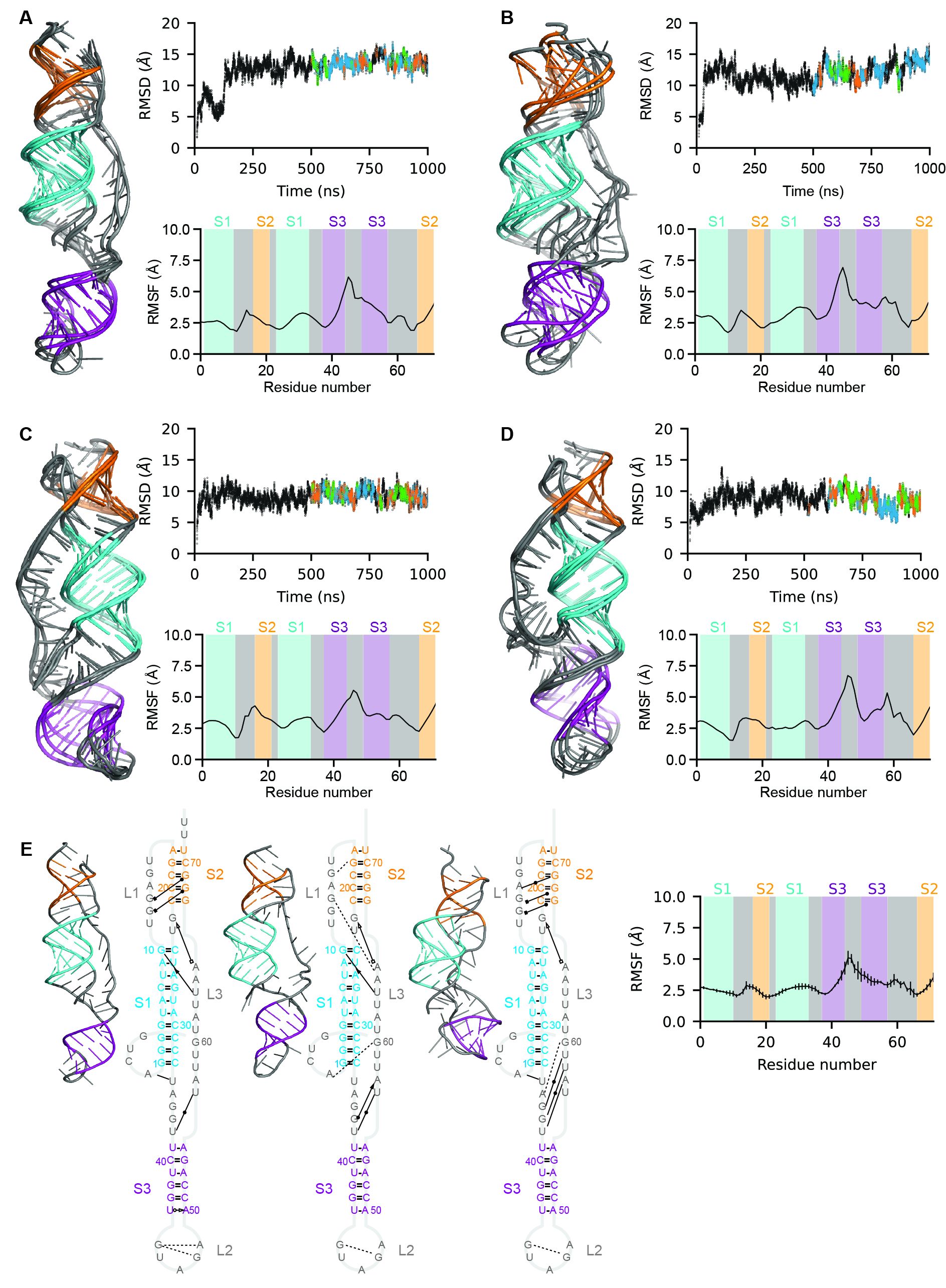

Supplement: S8 Fig — (A) Overlay of the 3D structures of the three most populated clusters from simulations of the 5′-threaded structure in Fig 3 without Mg2+. Cyan: S1, gold: S2, purple: S3. Top inset: RMSD vs time. Portions of this trajectory in which the top three clusters are occupied are indicated in color (cyan: cluster 1, orange: cluster 2, green: cluster 3). Bottom inset: RMSF for each residue. (B) The same for the 5′-threaded structure in Fig 3 with Mg2+ (ions not shown for clarity). (C) The same for the unthreaded structure in Fig 3 without Mg2+. (D) The same for the unthreaded structure in Fig 3 with Mg2+ (ions not shown for clarity). (E) The top cluster from replicate simulations starting from the 3 lowest-energy FARFAR2 predictions (lowest-energy on left), showing qualitatively similar results. Right: average RMSF over the replicates; error bars indicate s.e.m. (TIF) [file pcbi.1011124.s012.tif]

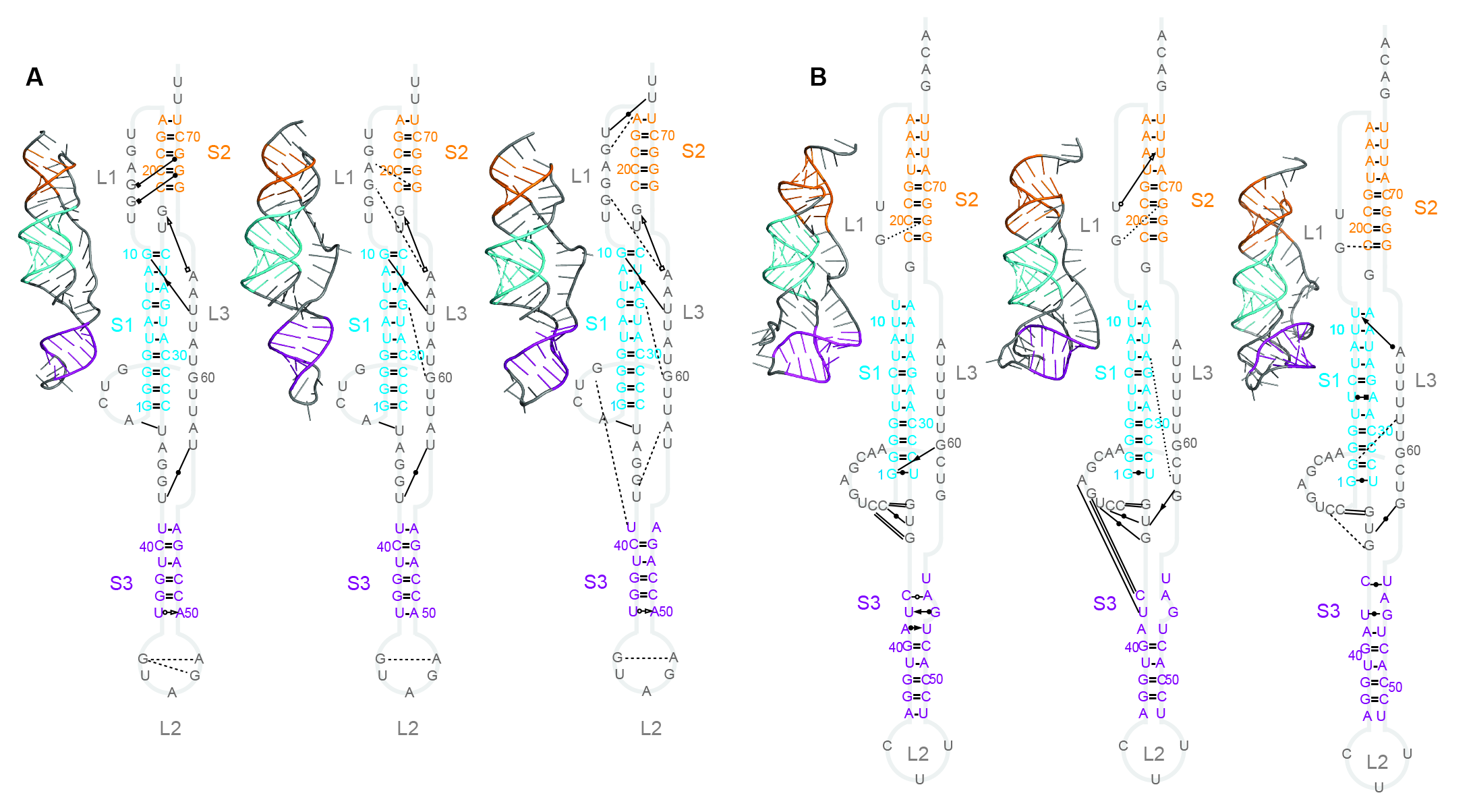

Supplement: S9 Fig — (A) Results for the most-occupied cluster of GLGC2 pseudoknot are qualitatively the same from 3 separate replicates. (B) Same for Vs-CoV-1 pseudoknot. (TIF) [file pcbi.1011124.s013.tif]

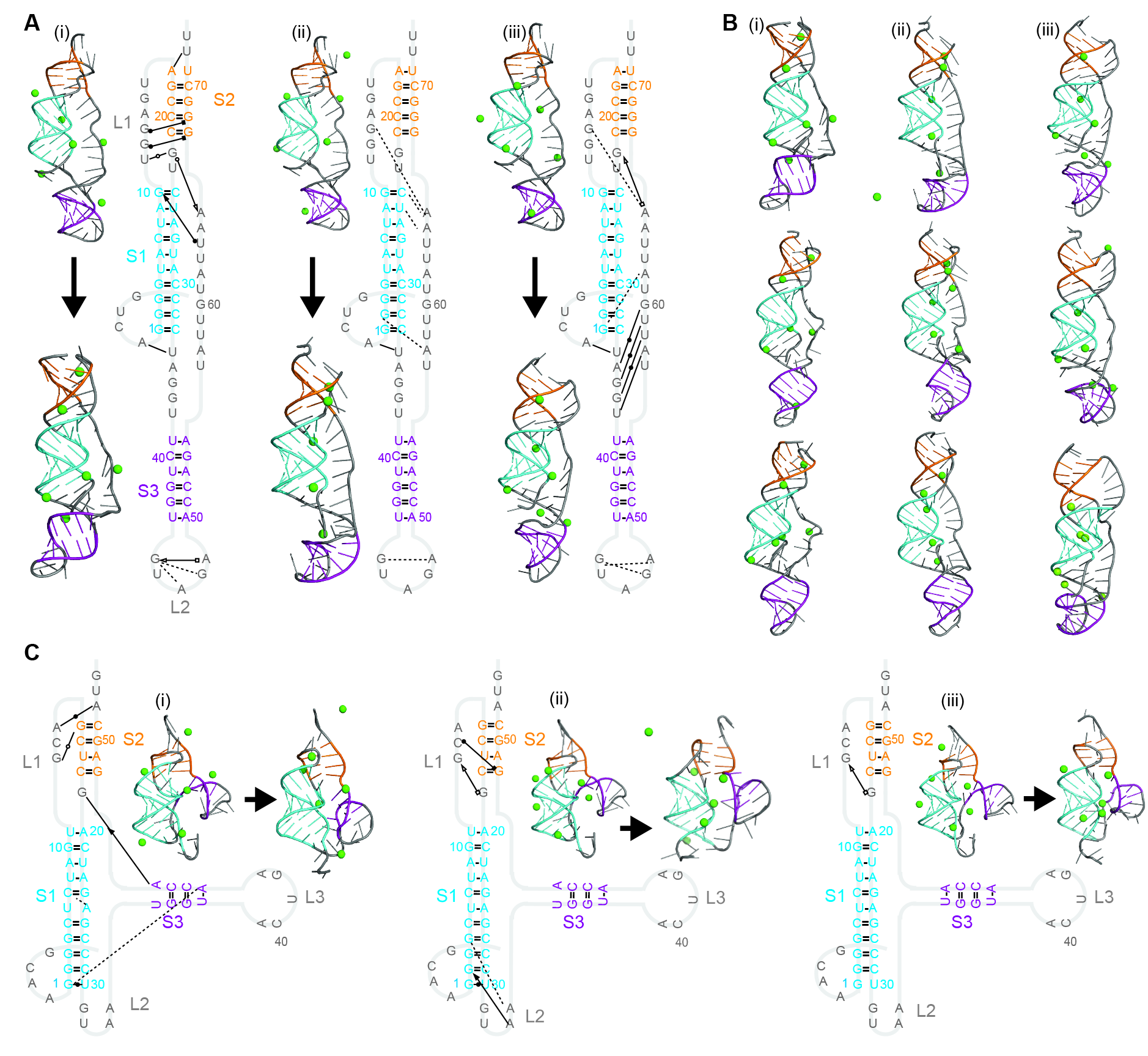

Supplement: S10 Fig — (A) Secondary structure, tertiary contacts, and 3D structure of the most-occupied cluster from MD simulations of the threaded conformer of the GLGC2 pseudoknot with Mg2+ ions initially placed at three different sets of positions (i–iii). Top: initial ion positions in predicted 3D structure; bottom: result after MD simulation. (B) 3D structures and ion positions for top 3 clusters for each of the 3 initial ion positions, showing ion positions are not fully equilibrated. (C) Secondary structure, tertiary contacts, and 3D structure of the most-occupied cluster from MD simulations of the threaded conformer of the Anlong-172 pseudoknot with Mg2+ ions initially placed at three different sets of positions (i–iii). (TIF) [file pcbi.1011124.s014.tif]

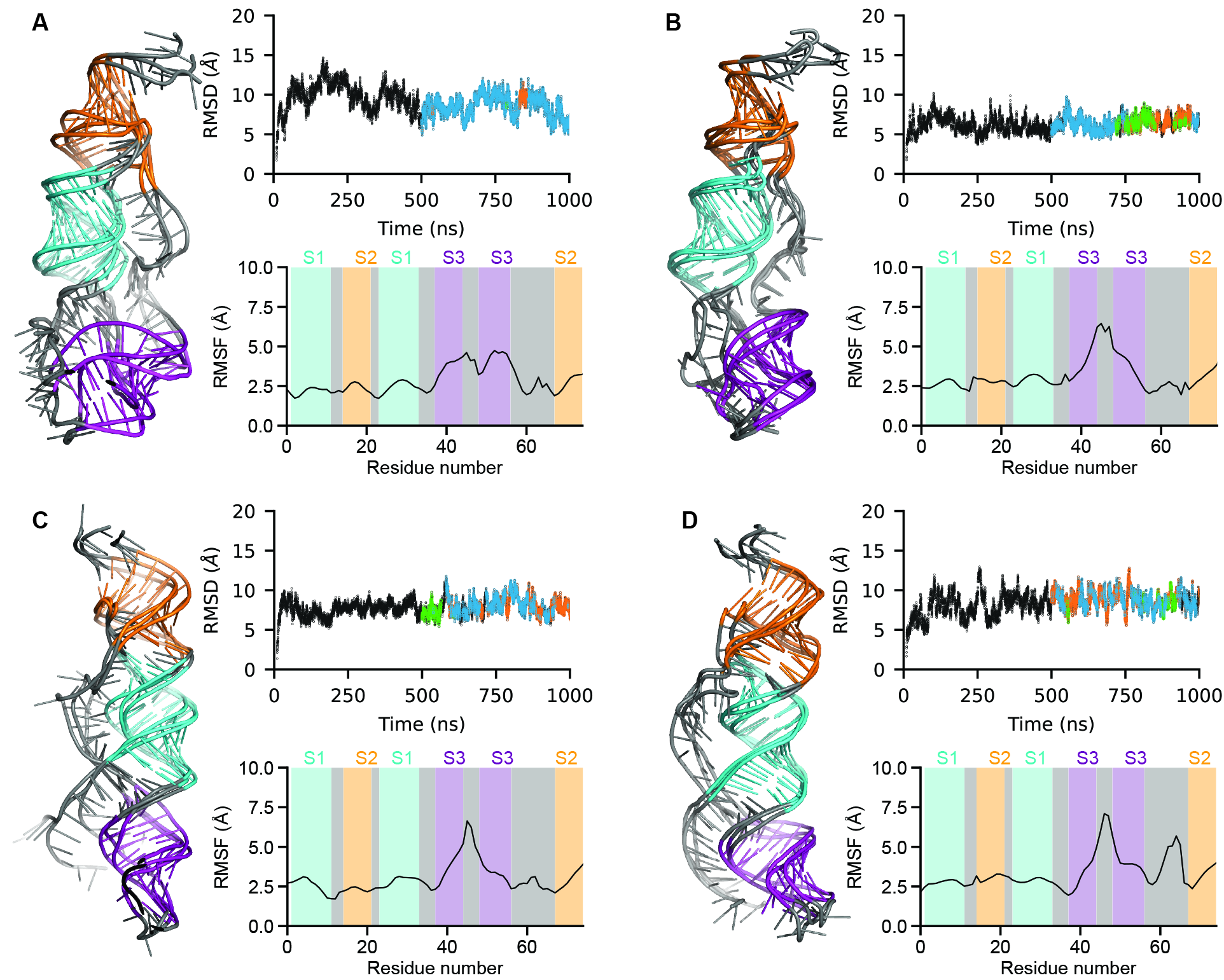

Supplement: S11 Fig — (A) Overlay of the 3D structures of the three most populated clusters from simulations of the 5′-threaded structure in Fig 4 without Mg2+. Cyan: S1, gold: S2, purple: S3. Top inset: RMSD vs time. Portions of this trajectory in which the top three clusters are occupied are indicated in color (cyan: cluster 1, orange: cluster 2, green: cluster 3). Bottom inset: RMSF for each residue. (B) The same for the 5′-threaded structure in Fig 4 with Mg2+ (ions not shown for clarity). (C) The same for the unthreaded structure in Fig 4 Mg2+. (D) The same for the unthreaded structure in Fig 4 with Mg2+ (ions not shown for clarity). (TIF) [file pcbi.1011124.s015.tif]

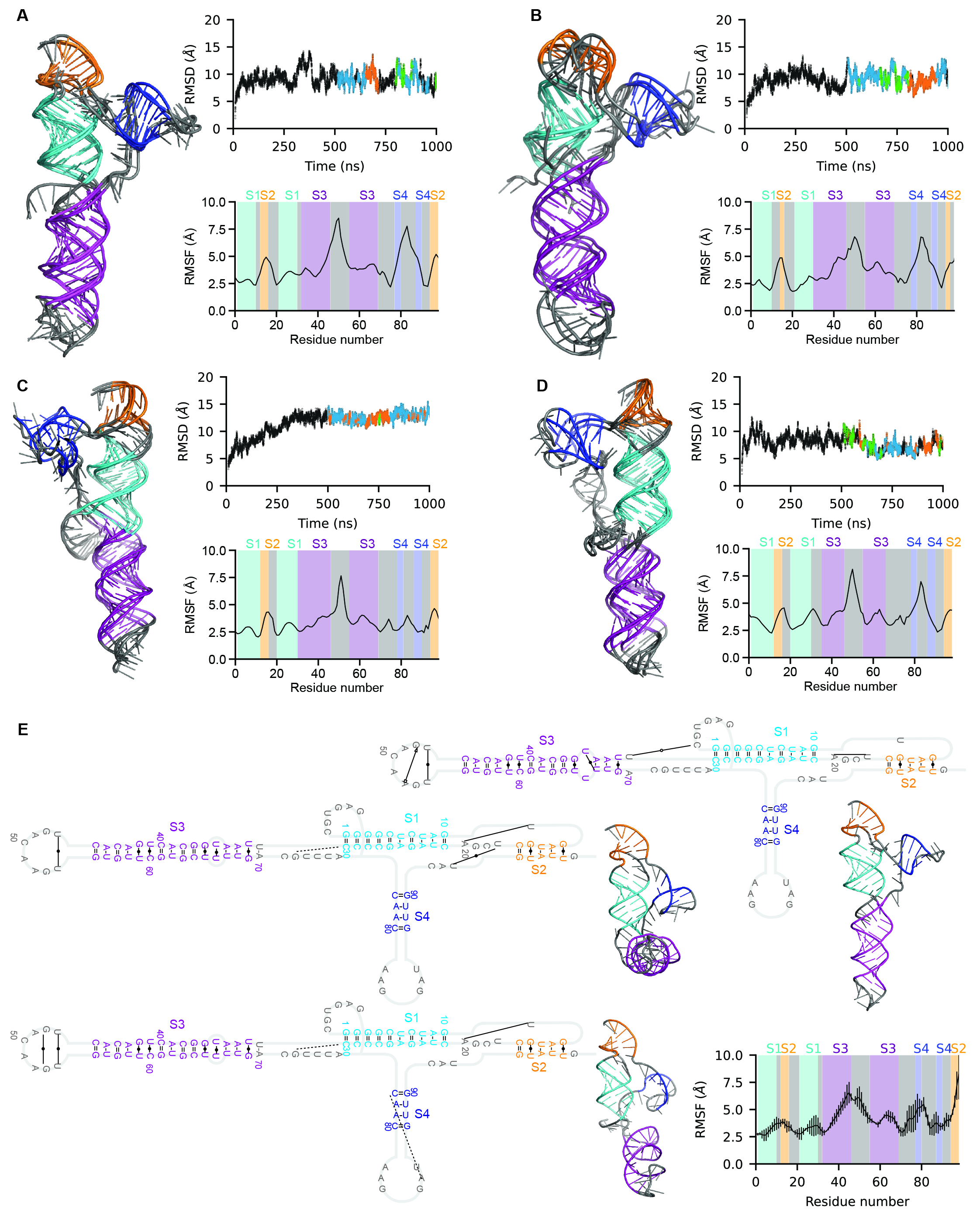

Supplement: S12 Fig — (A) Overlay of the 3D structures of the three most populated clusters from simulations of the 5′-threaded structure in Fig 5 without Mg2+. Cyan: S1, gold: S2, purple: S3, blue: S4. Top inset: RMSD vs time. Portions of this trajectory in which the top three clusters are occupied are indicated in color (cyan: cluster 1, orange: cluster 2, green: cluster 3). Bottom inset: RMSF for each residue. (B) The same for the 5′-threaded structure in Fig 5 with Mg2+ (ions not shown for clarity). (C) The same for the unthreaded structure in Fig 5 without Mg2+. (D) The same for the unthreaded structure in Fig 5 with Mg2+ (ions not shown for clarity). (E) Top cluster from replicate simulations starting from 3 lowest-energy FARFAR2 predictions (lowest-energy on top), showing qualitatively similar results with the exception of the orientation of S3. Right: average RMSF over 3 replicates; error bars indicate s.e.m. (TIF) [file pcbi.1011124.s016.tif]

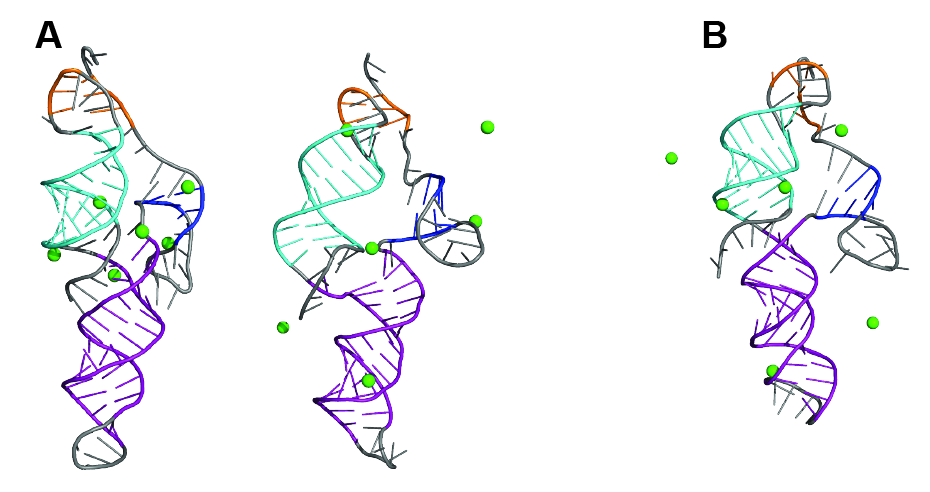

Supplement: S13 Fig — Replicates with Mg2+ ions at different initial positions show that S1/S3 stacking is disrupted if Mg2+ is bound at the S1/S3 interface (A), but preserved otherwise (B). (TIF) [file pcbi.1011124.s017.tif]

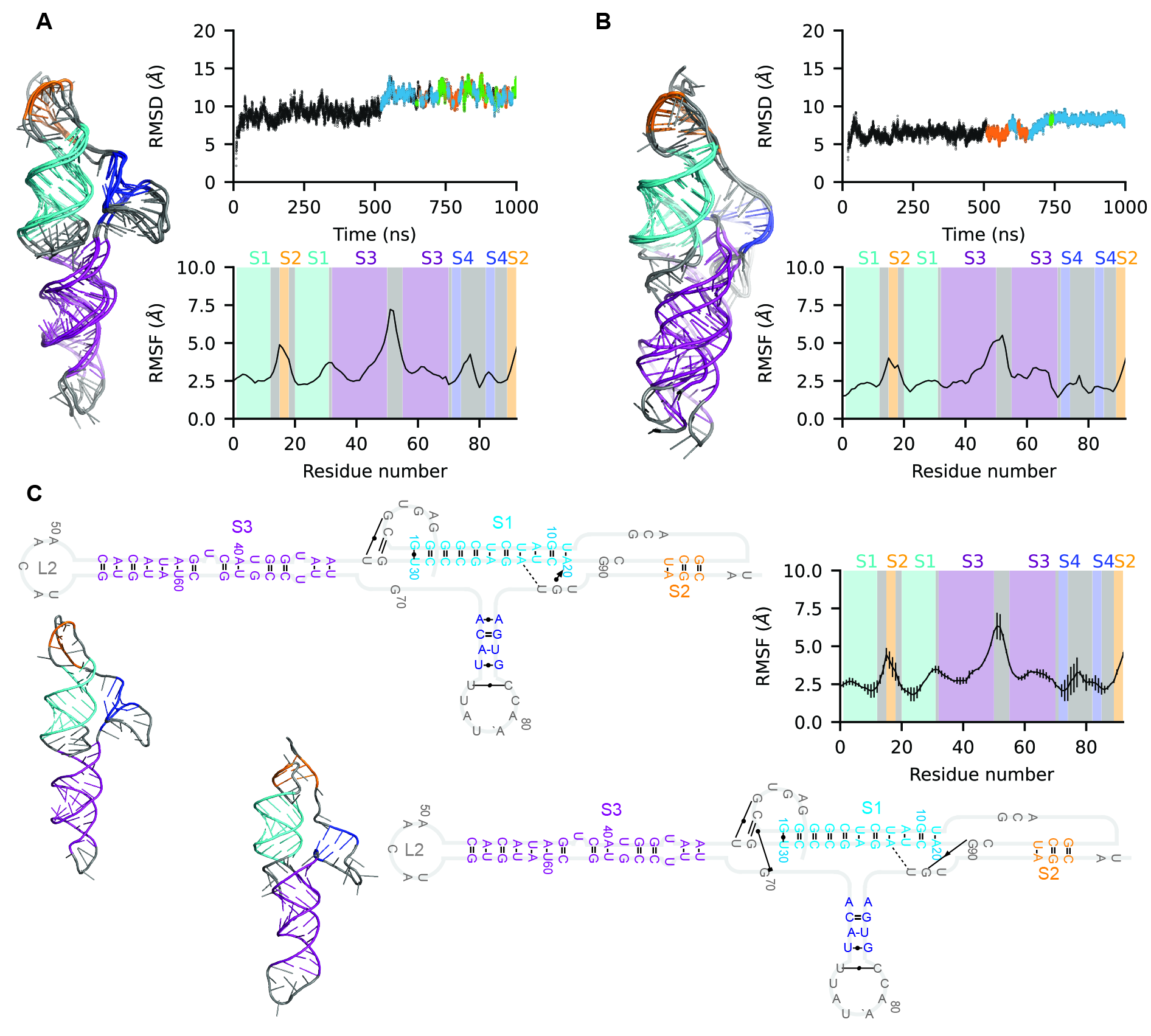

Supplement: S14 Fig — (A) Overlay of the 3D structures of the three most populated clusters from simulations of the 5′-threaded structure in Fig 6 without Mg2+. Cyan: S1, gold: S2, purple: S3, blue: S4. Top inset: RMSD vs time. Portions of this trajectory in which the top three clusters are occupied are indicated in color (cyan: cluster 1, orange: cluster 2, green: cluster 3). Bottom inset: RMSF for each residue. (B) The same for the 5′-threaded structure in Fig 6 with Mg2+ (ions not shown for clarity). (C) Top cluster from replicate simulations starting from lowest- and 3rd-lowest-energy FARFAR2 predictions (lowest-energy on top), showing qualitatively similar results; 2nd-lowest energy structure had a different fold that was not stable in MD simulation. Right: average RMSF over replicates. (TIF) [file pcbi.1011124.s018.tif]

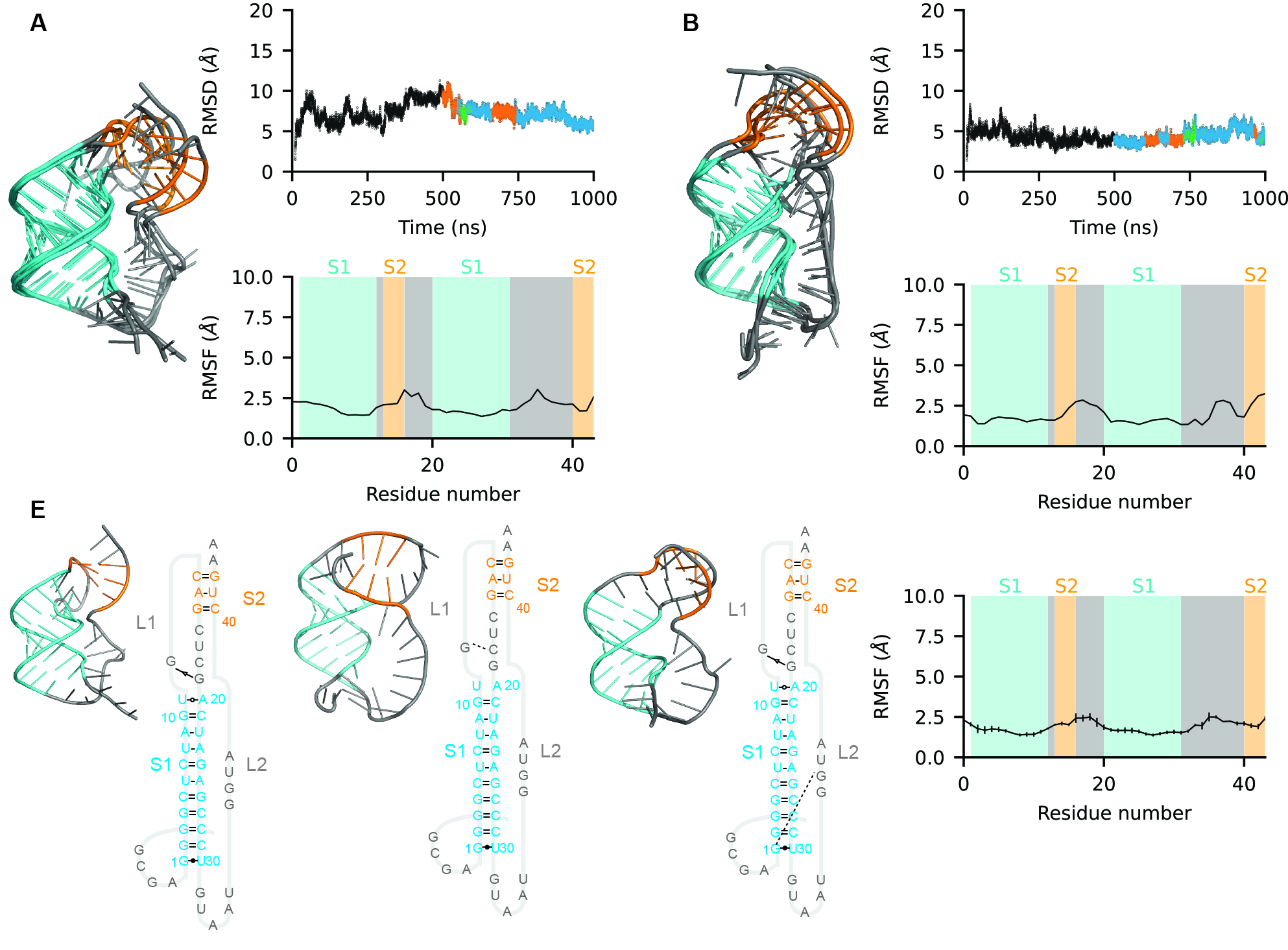

Supplement: S15 Fig — (A) Overlay of the 3D structures of the three most populated clusters from simulations of the 5′-threaded structure in Fig 7 without Mg2+. Cyan: S1, gold: S2, purple: S3, blue: S4. Top inset: RMSD vs time. Portions of this trajectory in which the top three clusters are occupied are indicated in color (cyan: cluster 1, orange: cluster 2, green: cluster 3). Bottom inset: RMSF for each residue. (B) The same for the 5′-threaded structure in Fig 7 with Mg2+ (ions not shown for clarity). (C) Top cluster from replicate simulations starting from 3 lowest-energy FARFAR2 predictions (lowest-energy on left), showing qualitatively similar results. Right: average RMSF over 3 replicates; error bars indicate s.e.m. (TIF) [file pcbi.1011124.s019.tif]

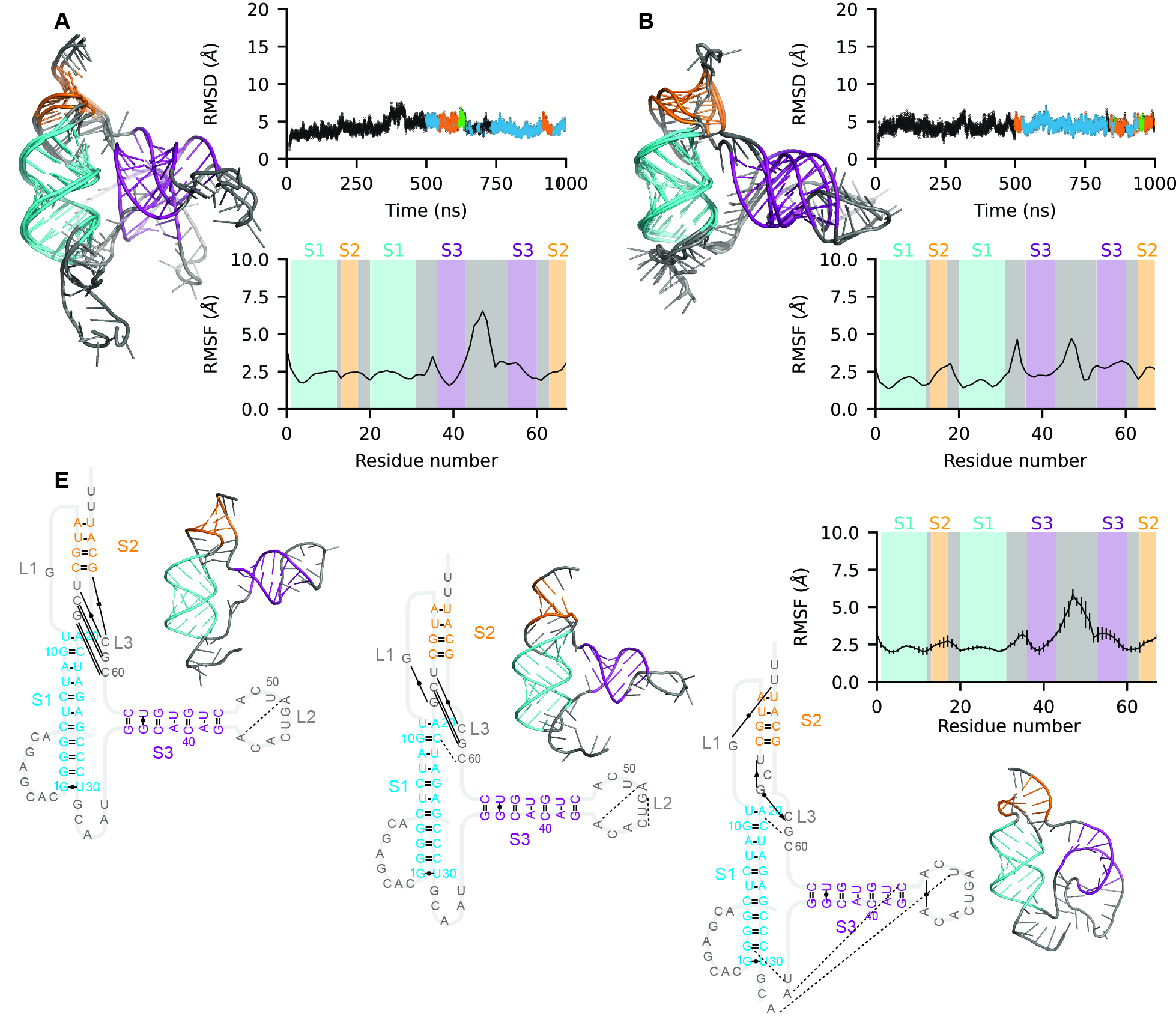

Supplement: S16 Fig — (A) Overlay of the 3D structures of the three most populated clusters from simulations of the 5′-threaded structure in Fig 8 without Mg2+. Cyan: S1, gold: S2, purple: S3, blue: S4. Top inset: RMSD vs time. Portions of this trajectory in which the top three clusters are occupied are indicated in color (cyan: cluster 1, orange: cluster 2, green: cluster 3). Bottom inset: RMSF for each residue. (B) The same for the 5′-threaded structure in Fig 8 with Mg2+ (ions not shown for clarity). (C) Top cluster from replicate simulations starting from 3 lowest-energy FARFAR2 predictions (lowest-energy on left), showing qualitatively similar results, with the exception of S3 orientation. Right: average RMSF over 3 replicates; error bars indicate s.e.m. (TIF) [file pcbi.1011124.s020.tif]

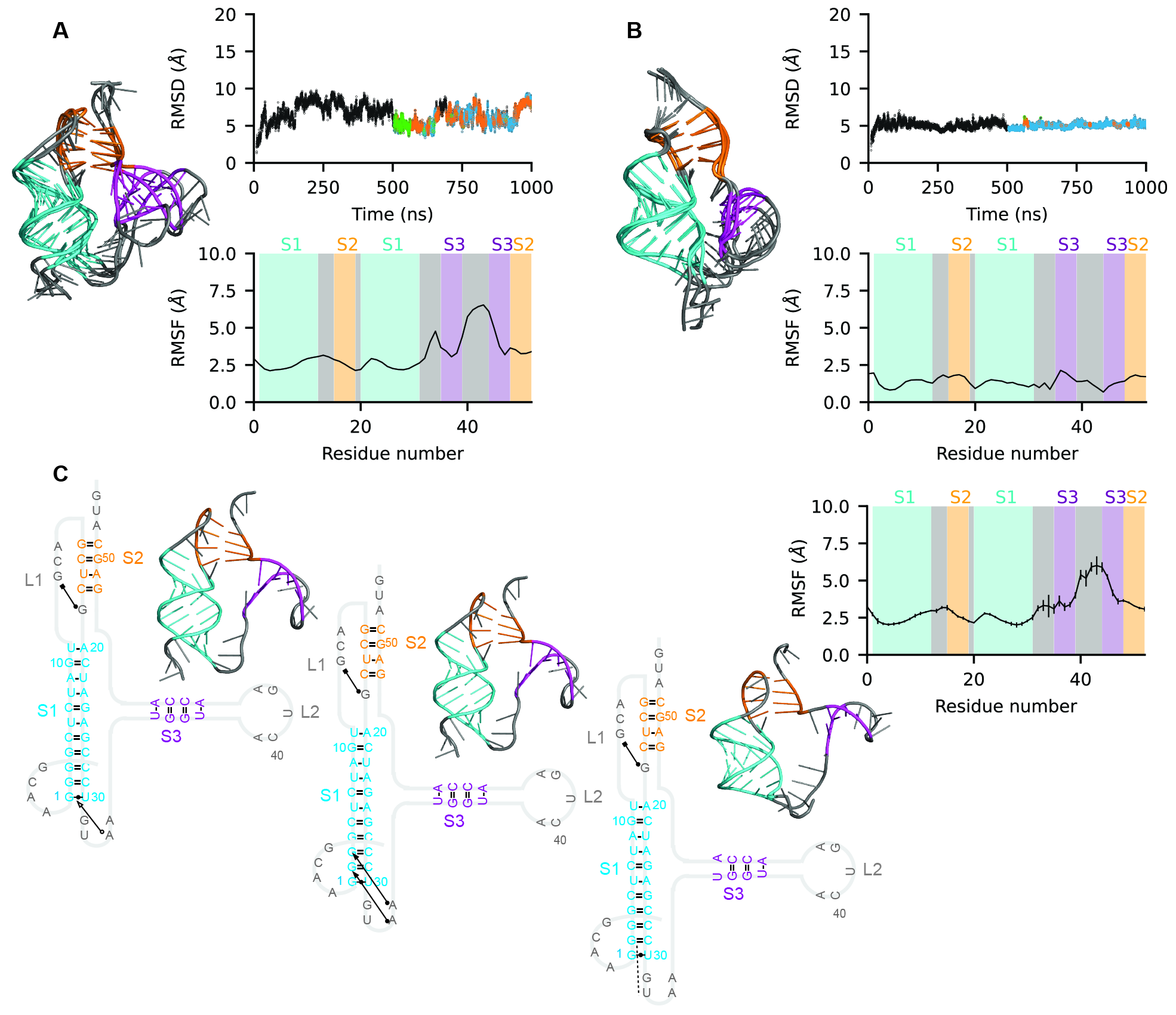

Supplement: S17 Fig — (A) Overlay of the 3D structures of the three most populated clusters from simulations of the 5′-threaded structure in Fig 9 without Mg2+. Cyan: S1, gold: S2, purple: S3, blue: S4. Top inset: RMSD vs time. Portions of this trajectory in which the top three clusters are occupied are indicated in color (cyan: cluster 1, orange: cluster 2, green: cluster 3). Bottom inset: RMSF for each residue. (B) The same for the 5′-threaded structure in Fig 9 with Mg2+ (ions not shown for clarity). (C) Top cluster from replicate simulations starting from 3 lowest-energy FARFAR2 predictions (lowest-energy on left), showing qualitatively similar results. Right: average RMSF over 3 replicates; error bars indicate s.e.m. (TIF) [file pcbi.1011124.s021.tif]

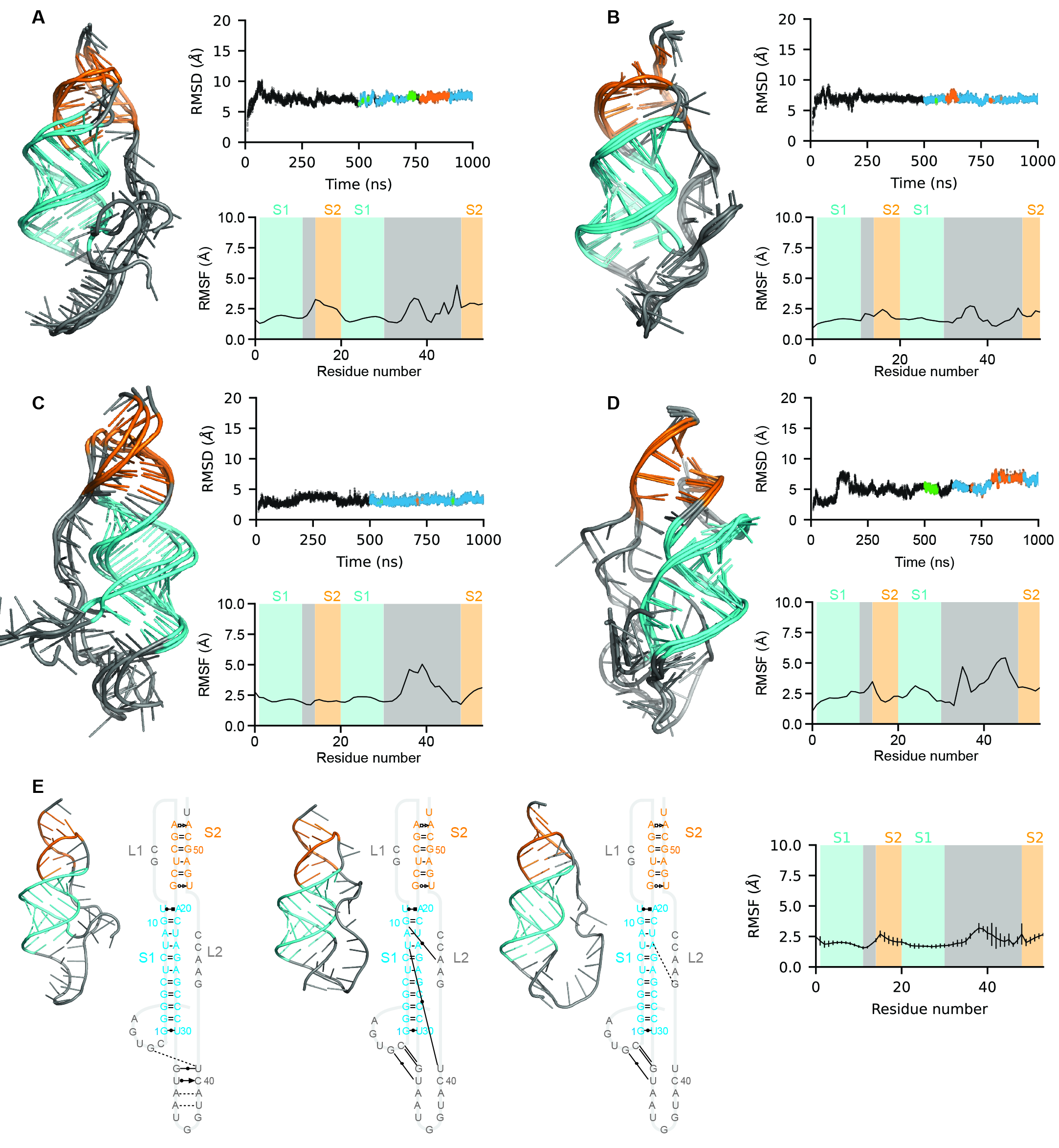

Supplement: S18 Fig — (A) Overlay of the 3D structures of the three most populated clusters from simulations of the 5′-threaded structure in Fig 10 without Mg2+. Cyan: S1, gold: S2. Top inset: RMSD vs time. Portions of this trajectory in which the top three clusters are occupied are indicated in color (cyan: cluster 1, orange: cluster 2, green: cluster 3). Bottom inset: RMSF for each residue. (B) The same for the 5′-threaded structure in Fig 10 with Mg2+ (ions not shown for clarity). (C) The same for the unthreaded structure in Fig 10 without Mg2+. (D) The same for the unthreaded structure in Fig 10 with Mg2+ (ions not shown for clarity). (E) Top cluster from replicate simulations starting from 3 lowest-energy FARFAR2 predictions (lowest-energy on left), showing qualitatively similar results, with the exception of L2. Right: average RMSF over 3 replicates; error bars indicate s.e.m. (TIF) [file pcbi.1011124.s022.tif]

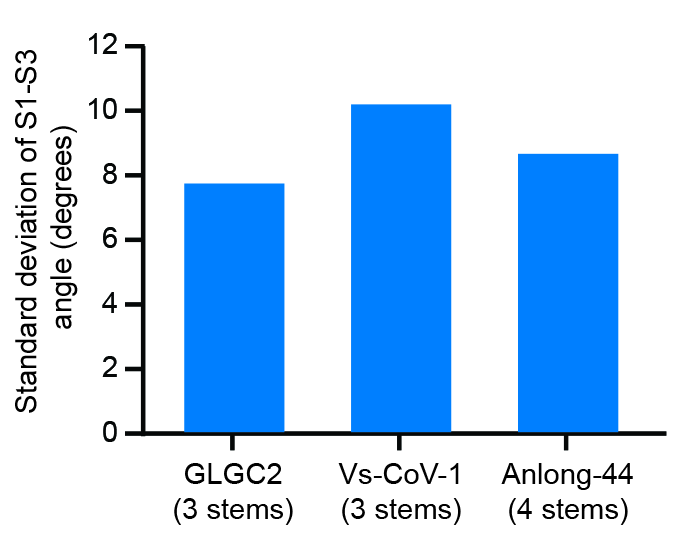

Supplement: S19 Fig — The S1-S3 angle was measured from 5,000 frames (one every 0.1 ns) of the threaded conformers without Mg2+ and the standard deviation calculated as a measure of the flexibility of S3, for the 3- and 4-stem pseudoknots whose frameshifting has been measured experimentally. (TIF) [file pcbi.1011124.s023.tif]
