# Supplementary material for: Modelling the structures of frameshift-stimulatory pseudoknots from representative bat coronaviruses
Source: PLoS Comput Biol. 2023 May 19;19(5):e1011124. doi: 10.1371/journal.pcbi.1011124 (PMC10234561; doi:10.1371/journal.pcbi.1011124)
Supplement: S1 Table — Consensus 2D structure predictions for all pseudoknots in Fig 1, in dot-bracket notation. Source viruses listed by NCBI Virus accession number. Pseudoknots selected as representative examples from each cluster for structural characterization colored in red. Predictors contributing to consensus shown by tick mark. Note that the consensus prediction for SARS-CoV-2 (accession number NC_045512) matches the secondary restructure reported in cryo-EM structures of the pseudoknot [18,21]. (DOC) [file pcbi.1011124.s004.doc]

**S1 Table. Secondary structure predictions**. Consensus 2D structure predictions for all pseudoknots in Fig 1, in dot-bracket notation. Source viruses listed by NCBI Virus accession number. Pseudoknots selected as representative examples from each cluster for structural characterization colored in red. Predictors contributing to consensus shown by tick mark. Note that the consensus prediction for SARS-CoV-2 (accession number NC_045512) matches the secondary restructure reported in cryo-EM structures of the pseudoknot [1,2].

| **Accession Number** | **Secondary Structure** |  | **Consensus prediction** | | | |
| --- | --- | --- | --- | --- | --- | --- |
| **Cluster 1** | |  | pKiss | Hotknots | PKNOTS | NUPACK |
| MG693172 | [[[[[[[[[[[.....{{{{{{]]]]]]]]]]].......((((.....))))............}}}}}} |  | ✓ | ✓ | 🗶 | 🗶 |
| MG693168 | [[[[[[[[[[[.....{{{{{{]]]]]]]]]]].......((((.....))))............}}}}}} |  | ✓ | ✓ | 🗶 | 🗶 |
| NC_048212 | [[[[[[[[[[[.....{{{{{{]]]]]]]]]]].......((((.....))))............}}}}}} |  | ✓ | ✓ | 🗶 | 🗶 |
| MG693169 | [[[[[[[[[[[.....{{{{{{]]]]]]]]]]].......((((.....))))............}}}}}} |  | ✓ | ✓ | 🗶 | ✓ |
| MG693171 | [[[[[[[[[[[.....{{{{{{]]]]]]]]]]].......((((.....))))............}}}}}} |  | ✓ | ✓ | 🗶 | ✓ |
| KU182958 | [[[[[[[[[[......{{{{{..]]]]]]]]]].....((((((.....))))))...........}}}}} |  | ✓ | ✓ | 🗶 | ✓ |
| KU182959 | [[[[[[[[[[......{{{{{..]]]]]]]]]].....((((((.....))))))...........}}}}} |  | ✓ | ✓ | 🗶 | ✓ |
| KU182960 | [[[[[[[[[[......{{{{{..]]]]]]]]]].....((((((.....))))))...........}}}}} |  | ✓ | ✓ | 🗶 | ✓ |
| MG693170 | [[[[[[[[[[......{{{{{..]]]]]]]]]].....((((((.....))))))...........}}}}} |  | ✓ | ✓ | 🗶 | ✓ |
| MK492263 | [[[[[[[[[[......{{{{{..]]]]]]]]]].....(((((.......)))))...........}}}}} |  | ✓ | ✓ | 🗶 | ✓ |
| KU182961 | [[[[[[[[[[......{{{{{..]]]]]]]]]].....(((((.......)))))...........}}}}} |  | ✓ | ✓ | 🗶 | ✓ |
| KU182962 | [[[[[[[[[[......{{{{{..]]]]]]]]]].....(((((.......)))))...........}}}}} |  | ✓ | ✓ | 🗶 | ✓ |
| **Cluster 2** | |  |  | | | |
| LC469308 | [[[[[[[[[[[..{{{{{{{{.]]]]]]]]]]]....(((((((...)))))))............}}}}}}}} |  | ✓ | ✓ | ✓ | ✓ |
| NC_034440 | [[[[[[[[[[[..{{{{{{{{.]]]]]]]]]]]..((((((((.....)))))..)))........}}}}}}}} |  | ✓ | ✓ | ✓ | ✓ |
| KX574227 | [[[[[[[[[[[..{{{{{{{{.]]]]]]]]]]]..((((((((.....)))))..)))........}}}}}}}} |  | ✓ | ✓ | ✓ | ✓ |
| KU182965 | [[[[[[[[[[[...{{{{{{{.]]]]]]]]]]]..(((.(((((...))))))))...........}}}}}}} |  | ✓ | ✓ | ✓ | ✓ |
| KU182956 | [[[[[[[[[[[...{{{{{{{.]]]]]]]]]]]..(((.(((((...))))))))...........}}}}}}} |  | ✓ | ✓ | ✓ | ✓ |
| KU182957 | [[[[[[[[[[[...{{{{{{{.]]]]]]]]]]]..(((.(((((...))))))))...........}}}}}}} |  | ✓ | ✓ | ✓ | ✓ |
| **SARS-like cluster** | |  |  | | | |
| MT726044 | [[[[[[[[[[...{{{{{..]]]]]]]]]](((((((((.........))).)))))).....}}}}} |  | ✓ | ✓ | ✓ | 🗶 |
| NC_045512 | [[[[[[[[[[...{{{{{..]]]]]]]]]](((((((((.........))).)))))).....}}}}} |  | ✓ | ✓ | ✓ | ✓ |
| MW201981 | [[[[[[[[[[...{{{{{..]]]]]]]]]](((((((((.........))).)))))).....}}}}} |  | ✓ | ✓ | ✓ | ✓ |
| MZ937004 | [[[[[[[[[[...{{{{{..]]]]]]]]]](((((((((.........))).)))))).....}}}}} |  | ✓ | ✓ | ✓ | ✓ |
| MZ937003 | [[[[[[[[[[...{{{{{..]]]]]]]]]](((((((((.........))).)))))).....}}}}} |  | ✓ | ✓ | ✓ | ✓ |
| MZ937002 | [[[[[[[[[[...{{{{{..]]]]]]]]]](((((((((.........))).)))))).....}}}}} |  | ✓ | ✓ | ✓ | ✓ |
| MZ937000 | [[[[[[[[[[...{{{{{..]]]]]]]]]](((((((((.........))).)))))).....}}}}} |  | ✓ | ✓ | ✓ | ✓ |
| MZ937001 | [[[[[[[[[[...{{{{{..]]]]]]]]]](((((((((.........))).)))))).....}}}}} |  | ✓ | ✓ | ✓ | ✓ |
| **Cluster 3** | |  |  | | | |
| MN535734 | [[[[[[[[[[[......{{]]]]]]]]]]].....(((((..(((((....)))))....))))).....}} |  | ✓ | 🗶 | 🗶 | 🗶 |
| KY770857 | [[[[[[[[[[[.{{{{...]]]]]]]]]]]((((.(((((.(((((........))))).))))).))))......(((((....)))))...}}}} |  | ✓ | 🗶 | 🗶 | 🗶 |
| KY770856 | [[[[[[[[[[[.{{{{...]]]]]]]]]]]((((.(((((.(((((........))))).))))).))))......(((((....)))))...}}}} |  | ✓ | 🗶 | 🗶 | 🗶 |
| KY770854 | [[[[[[[[[[[.{{{{...]]]]]]]]]]]((((.(((((.(((((........))))).))))).))))......(((((....)))))...}}}} |  | ✓ | 🗶 | 🗶 | 🗶 |
| KY770850 | [[[[[[[[[[[.{{{{...]]]]]]]]]]]((((.(((((.(((((........))))).))))).))))......(((((....)))))...}}}} |  | ✓ | 🗶 | 🗶 | 🗶 |
| KY770851 | [[[[[[[[[[[.{{{{...]]]]]]]]]]]((((.(((((.(((((........))))).))))).))))......(((((....)))))...}}}} |  | ✓ | 🗶 | 🗶 | 🗶 |
| **Cluster 4** | |  |  |  |  |  |
| MN482242 | [[[[[[[[[[[..{{{{{{]]]]]]]]]]](((.((((.(((...))))))))))....}}}}}} |  | ✓ | 🗶 | 🗶 | ✓ |
| MZ218060 | [[[[[[[[[[.{{{{{{...]]]]]]]]]].....(((((.((((((((...)))))))))))))........((((....))))}}}}}} |  | ✓ | 🗶 | 🗶 | ✓ |
| MN535733 | [[[[[[[[[[[..{{{...]]]]]]]]]]]..........(((((((((...))))))))).}}} |  | ✓ | 🗶 | 🗶 | 🗶 |
| MN482243 | [[[[[[[[[[[...{{{..]]]]]]]]]]].(((.(((((.((((((((...))))))))))))).))).(((........)))....}}} |  | ✓ | 🗶 | 🗶 | 🗶 |
| MZ218052 | [[[[[[[[[[[...{{{..]]]]]]]]]]].(((.(((((.((((((((...))))))))))))).))).(((........)))....}}} |  | ✓ | 🗶 | 🗶 | 🗶 |
| MN535731 | [[[[[[[[[[[...{{{..]]]]]]]]]]].(((.(((((.((((((((...))))))))))))).))).(((........)))....}}} |  | ✓ | 🗶 | 🗶 | 🗶 |
| MN535732 | [[[[[[[[[[[...{{{..]]]]]]]]]]].(((.(((((.((((((((...))))))))))))).))).(((........)))....}}} |  | ✓ | 🗶 | 🗶 | 🗶 |
| **Cluster 5** | |  |  |  |  |  |
| KU182955 | [[[[[[[[[[[.{{{....]]]]]]]]]]].........}}} |  | ✓ | 🗶 | 🗶 | 🗶 |
| KU182954 | [[[[[[[[[[[.{{{....]]]]]]]]]]].........}}} |  | ✓ | 🗶 | 🗶 | 🗶 |
| KF294268 | [[[[[[[[[[[.{{{....]]]]]]]]]]].........}}} |  | ✓ | 🗶 | 🗶 | 🗶 |
| KF294269 | [[[[[[[[[[[.{{{....]]]]]]]]]]].........}}} |  | ✓ | 🗶 | 🗶 | 🗶 |
| KF294270 | [[[[[[[[[[[.{{{....]]]]]]]]]]].........}}} |  | ✓ | 🗶 | 🗶 | 🗶 |
| KF294271 | [[[[[[[[[[[.{{{....]]]]]]]]]]].........}}} |  | ✓ | 🗶 | 🗶 | 🗶 |
| KF294275 | [[[[[[[[[[[.{{{....]]]]]]]]]]].........}}} |  | ✓ | 🗶 | 🗶 | 🗶 |
| KF294277 | [[[[[[[[[[[.{{{....]]]]]]]]]]].........}}} |  | ✓ | 🗶 | 🗶 | 🗶 |
| **Cluster 6** | |  |  |  |  |  |
| OL405717 | [[[[[[[[[[[.{{.....]]]]]]]]]]].....(((((.((((((......)))))).))))).............}} |  | ✓ | ✓ | 🗶 | 🗶 |
| MG916904 | [[[[[[[[[.{{{{...]]]]]]]]].(((...(((((.((((((......)))))).)))))....))).................}}}} |  | ✓ | 🗶 | 🗶 | 🗶 |
| MG916901 | [[[[[[[[[[[...{{{..]]]]]]]]]]].(((..((((.(((((........))))).))))..)))...................}}} |  | ✓ | 🗶 | 🗶 | 🗶 |
| MG916903 | [[[[[[[[[[[.{{{{...]]]]]]]]]]].....(((((((..........)))))))...}}}} |  | ✓ | ✓ | 🗶 | 🗶 |
| OL405715 | [[[[[[[[[[[.{{{{...]]]]]]]]]]].....(((((((..........)))))))...}}}} |  | ✓ | ✓ | 🗶 | 🗶 |
| OL405716 | [[[[[[[[[[[.{{{{...]]]]]]]]]]].....(((((((..........)))))))...}}}} |  | ✓ | ✓ | 🗶 | 🗶 |
| OL405714 | [[[[[[[[[[[.{{{{...]]]]]]]]]]].....(((((((..........)))))))...}}}} |  | ✓ | ✓ | 🗶 | 🗶 |
| MG916902 | [[[[[[[[[[[.{{{{...]]]]]]]]]]].....(((((((..........)))))))...}}}} |  | ✓ | ✓ | 🗶 | 🗶 |
| **Cluster 7** | |  |  |  |  |  |
| KF294279 | [[[[[[[[[[[...{{{{.]]]]]]]]]]]....((((.....))))}}}} |  | 🗶 | ✓ | ✓ | ✓ |
| KF294281 | [[[[[[[[[[[...{{{{.]]]]]]]]]]]....((((.....))))}}}} |  | 🗶 | ✓ | ✓ | ✓ |
| KF294280 | [[[[[[[[[[[...{{{{.]]]]]]]]]]]....((((.....))))}}}} |  | 🗶 | ✓ | ✓ | ✓ |
| KF294278 | [[[[[[[[[[[...{{{{.]]]]]]]]]]]....((((.....))))}}}} |  | 🗶 | ✓ | ✓ | ✓ |
| KF294276 | [[[[[[[[[[[...{{{{.]]]]]]]]]]]....((((.....))))}}}} |  | 🗶 | ✓ | ✓ | ✓ |
| KF294273 | [[[[[[[[[[[...{{{{.]]]]]]]]]]]....((((.....))))}}}} |  | 🗶 | ✓ | ✓ | ✓ |
| **Cluster 8** | |  |  |  |  |  |
| KF294282 | [[[[[[[[[[[...{{{{{]]]]]]]]]]].....(((.....)))}}}}} |  | 🗶 | ✓ | ✓ | ✓ |
| KU182966 | [[[[[[[[[[[.{{{....]]]]]]]]]]].........}}} |  | ✓ | 🗶 | 🗶 | 🗶 |
| MW249018 | [[[[[[[[[[[.{{{....]]]]]]]]]]].........}}} |  | ✓ | 🗶 | 🗶 | 🗶 |

References:

1. Bhatt PR, Scaiola A, Loughran G, Leibundgut M, Kratzel A, Meurs R, et al. Structural basis of ribosomal frameshifting during translation of the SARS-CoV-2 RNA genome. Science 2021; 372:1306–13.

2. Zhang K, Zheludev IN, Hagey RJ, Haslecker R, Hou YJ, Kretsch R, et al. Cryo-EM and antisense targeting of the 28-kDa frameshift stimulation element from the SARS-CoV-2 RNA genome. Nat. Struct. Mol. Biol. 2021; 28:747–54.
