# Supplementary material for: Modelling the structures of frameshift-stimulatory pseudoknots from representative bat coronaviruses
Source: PLoS Comput Biol. 2023 May 19;19(5):e1011124. doi: 10.1371/journal.pcbi.1011124 (PMC10234561; doi:10.1371/journal.pcbi.1011124)
Supplement: Attachment — Submitted filename: PLOS Review.docx [file pcbi.1011124.s024.docx]

**Review of the manuscript “Modelling the structures of frameshift-stimulatory pseudoknots from representative bat coronaviruses”**

In this manuscript, the authors build microsecond molecular dynamics simulations for frameshifting elements (FSEs) of 4 bat coronaviruses. Different initial 3D models are used, and simulations with and without Mg^2+^ are both performed. The two beta-CoVs display similar 3-stem H-type pseudoknots as SARS-CoV-2, while the two alpha-CoVs have a 4-stem and a 2-stem pseudoknot structure. Despite this difference, all FSEs adopt both 5’ end threading and non-threading topologies, suggesting the FSE structural heterogeneity of bat-CoVs.

Despite the potential utility for future drug targeting against bat-CoVs, more explanations are needed for the Methods section, and deeper analysis can be added to the Results and Discussion sections to contribute more therapeutic insights. Overall, the manuscript requires major revisions to be considered for publication. Below are the detailed comments:

1) In Methods, subsection “Bat-CoV pseudoknot sequences”, the authors perform clustering for 48 bat-CoV sequences. How exactly are the “pairwise genetic distances” calculated and clustered? Do the 5 clusters correspond to different subgenera? Moreover, in Fig.1, only 25 sequences are displayed. What happen to the other sequences, do they form different clusters?
2) In Methods, subsection “Blind structure prediction”, can the authors briefly explain why they select HotKnots and pKiss for 2D structure prediction and FARFAR2 for 3D prediction, and why these programs are enough?
3) In Methods, subsection “Molecular dynamics simulations”, can the authors give more details on how they place the Mg^2+^ ions, and is there any rational behind? Placing Mg^2+^ can be tricky as their placement could bias the simulation.
4) In the first paragraph of Results, the authors choose to model the 100-nt FSE sequences “starting 6 nt downstream of the UUUAAAC slippery sequence”. Why not include the slippery site and the spacer region? Also, “an additional 3-4 nt were added to the 5’ end of each RNA” after the 2D structure predictions. Why not add these nucleotides during 2D predictions?
5) In the second paragraph of Results, the authors “selected one example from each group as the initial structures for MD simulations”. How exactly are these initial structures selected?
6) During the 3D structure prediction and MD simulation, has the 2D structure folding changed? The authors can track the base pairing of different stems to help show the stabilization/convergence of their MD models.
7) The authors observe that non-threaded FSE structures have higher RMSF in the Loop 3 region when Mg^2+^ ions are present. Why is this the case, shouldn’t Mg^2+^ helps stabilizing the structure? Does this suggest the placement of Mg^2+^ can be improved?
8) The authors mention several times about the bulges in stems. What is the significance of this?
9) The authors mention the SARS-CoV-2 FSE structure many times in the manuscript. A supplementary figure can be provided for this structure, highlighting features like the threaded ring conformation.
10) In Discussion, the authors discuss the impact of S3 on stimulating -1 PRF. Exact frameshifting efficiency percentages can be provided for different viruses.
11) In Discussion, the authors suggest that “S4 may reduce -1 PRF by interfering sterically with contacts between the pseudoknot and ribosome, or with the ability of S3 to bend with respect to S1/S2”. Have the authors checked the dominant motions of different bat-CoV FSE trajectories? Does KY770854 trajectory show lack of S3 bending compared to the others?
12) In Discussion, the authors state “these structural similarities between pseudoknots representative of the different varieties found in bat CoVs suggest that it should be possible to find small-molecule ligands that can inhibit -1 PRF in a broad spectrum of CoVs”. Can the authors suggest specific FSE target regions based on the structural similarities?
13) In Discussion, the authors cite that some PRF inhibitors demonstrate different effects for beta-CoVs and alpha-CoVs, while some have similar effects for both. Can the authors elaborate more on this, by checking the binding pockets of the different inhibitors and relating with the structural differences between beta and alpha CoVs?
14) Throughout the manuscript, the authors use accession numbers instead of virus names. I often need to check backwards to see which accession number is for which virus, and is it beta or alpha coronavirus. The authors should improve this by giving the virus names as well.
15) For all 3D structure plots, the authors should label the 5’ and 3’ ends, as well as the stems.
