# Supplementary material for: Modelling the structures of frameshift-stimulatory pseudoknots from representative bat coronaviruses
Source: PLoS Comput Biol. 2023 May 19;19(5):e1011124. doi: 10.1371/journal.pcbi.1011124 (PMC10234561; doi:10.1371/journal.pcbi.1011124)
Supplement: Attachment — Submitted filename: Sekar_etal_Response to reviewer comments_SecondRev.doc [file pcbi.1011124.s026.doc]

**Response to reviewer comments**

**Reviewer 1**

*The authors have performed extensive revision and successfully addressed all my concerns with additional simulation replica and clarification on previous conclusions. The authors revisited the multiple sequence alignment and performed covariation analysis according to reviewers’ comments. RNA secondary structure predictions were examined with additional pseudoknot prediction softwares and more 3D structures were selected for MD simulations to validate the simulation reliability and confirm the structural features. The authors also performed simulations starting from the same RNA structure with different Mg2+ ions positions and updated the discussion. The links between pseudoknot sequences and structures are much clearer, and the MD simulation results are much more solid than what was presented initially*.

We thank the reviewer for the positive comments.

**Reviewer 2**

*The authors have done a great job addressing all my comments. The manuscript is much clearer now. Glad to see more extensive analysis of sequence alignment and structure prediction. Though this manuscript provides qualitative features rather than quantitative measurements of different coronavirus FSEs, it is still important for future antiviral therapeutic studies. Look forward to the authors’ future work addressing interesting questions like the varying PRF inhibitors’ effects.*

We thank the reviewer for the positive comments and recognition of the value of a qualitative approach.

**Reviewer 3**

*All my concerns and questions were sufficiently addressed. From my side the article is ready for publication.*

**Reviewer 4**

*I appreciate that the authors made substantial efforts to address reviewers’ concerns. In particular, the repetition of simulations provides additional information to address concerns of convergence and repeatability. Additional details for the conservation of secondary structure are also important*. *Overall, I appreciate the importance of the work. Excellent modeling methods were used.*

We thank the reviewer for these comments.

[Numbering added for convenience in the remainder of the document]

*[1.] I am still somewhat concerned that the manuscript is qualitative in its description of the results. There are missed opportunities to be more quantitative. For example, given replicates of simulations, 2D RMSD plots would assess the similarity of conformations sampled across the structures. Additionally, the extent to which base pairs are preserved in the calculations could be quantified.*

To address the reviewer’s concern, we have added the requested analyses. For each representative bat-CoV pseudoknot, we generated 2D RMSD plots between the equilibrated part of the trajectories for all replicates. These results have been included as Figure S5 in the revised manuscript. We also analyzed the extent of preservation for the base-pairs in the pseudoknot stems (after equilibration), finding that they were all preserved for at least 98.5% of time. We have revised the manuscript to mention this analysis (Results, p. 11–12). We nevertheless reiterate our contention that, in the absence of any experimental information about bat-CoV pseudoknot structures that could help to anchor deeper computational analysis, a largely qualitative approach is the most meaningful way to address the questions that are the focus of our work.

*There are still some details that must be provided to fully evaluate and reproduce the work:*

*[2.] First, infernal is stated to be used to align the sequences. My understanding is that infernal requires a covariance model to function, therefore there must have been an alignment that was made first to train the covariance model. The detailed steps need to be clear. Even if there is a program in infernal that aligns de novo and I am wrong, infernal is a large collection of tools and therefore the details should be specified.*

For the Infernal analysis, we built a covariance model using SARS-CoV-2 frameshift signal, as stated in the Methods section when discussing the sequence alignment and clustering. We used the SARS-CoV-2 frameshift signal, as done in one of the references suggested by reviewer 4 in the last round of review, because there is no experimental data on any bat-CoV frameshift signal secondary structure. The approach we took was similar to that in the reference mentioned in the Methods section (reference [27]): we used cmbuild to build the covariance model, which was then calibrated using cmcalibrate before using cmsearch with ‘-A’ option to output an MSA.

We have revised the manuscript to mention these details (Methods, p. 5).

*[3.] Figure S1 shows the dot-bracket representation of the structure, but does not show the nucleotide identities. The alignment should show both the nucleotides and the base pairing.*

Table S1 displays only the dot-bracket structure representation, for two reasons: (1) The purpose of this table is to show—in a way that is easily parsed visually—that the predicted secondary structures for each of the bat-CoV pseudoknots within a given cluster are very similar. We feel that adding the sequence information would detract from this purpose. (2) The sequence information is already provided in the multiple sequence alignment file, hence repeating it in Table S1 would be redundant.

Nevertheless, we appreciate that it would be useful to connect the sequence and dot-bracket structure more clearly. We have therefore created an additional Excel file (Data S2) that pairs the sequence and dot-bracket structure for each pseudoknot, using an equal-spaced font so that the sequence and dot-bracket representation are aligned visually.

*[4.] Six magnesium ions were added to each magnesium-containing simulation. The manuscript should specify how the exact location was chosen. Also, the manuscript might comment on the extent to which they move from their starting location during the simulations.*

As stated in the Methods section, the Mg^2+^ ions were placed manually in the structure, within several general areas specified in the Methods section. The placement was done in MOE. While we did include a figure in Supporting Information showing the initial placement of ions in the simulations for which we did multiple replicates (Fig. S7 in the first revision), we did not include a figure showing the initial placements for each of the simulations in the main text. We have now added such a figure (Figure S4 in the second revision). We have also revised the manuscript to clarify that ions were placed manually in MOE (Methods, p. 6).

Regarding the motion of Mg^2+^ ions during the simulation, we computed the root-mean-square fluctuations in the Mg^2+^ coordinates and found that the ions bound to the pseudoknot underwent maximum fluctuations of ~7 Å. Given that the ion positions are not fully equilibrated (as discussed in the last round of reviews and in the first revision, p. 12-13 and Fig. S7), however, it is unclear how meaningful this analysis is. We have revised the manuscript to mention this result (Results, p. 11).

*[5.] For the clustering of simulation snapshots, there are additional details that need to be provided. Please clarify whether the RMSDs were calculated using mass weighting, heavy atoms, or all-atoms with no weights. Please also specify if the clustering was done with multiple trajectories concatenated or whether each trajectory was clustered by itself. The supplementary figures color annotate the clusters as a function of time. The colors are repeated, so I can hypothesize the clusters are the same (from concatenating) or that the coloring is just repeated. (In my opinion, either approach is fine; I just want to know what was done.)*

The trajectories were clustered individually (not concatenated). Clustering was done with the hierarchical agglomerative approach using mass-weighted RMSD of non-hydrogen backbone atoms, ignoring the flexible regions. We have revised the Methods section (p. 7) to clarify these points.

The RMSD plots provided in the Supporting Figures for each bat-CoV pseudoknot are from the trajectories starting with the top-ranked structure from farfar2 (not concatenated trajectories from replicates). We did not show RMSD plots for replicate simulations because the panels would become very busy while providing very little additional information. The top 3 clusters within the trajectory are indicated by color in each plot; the colors are repeated in many plots because the pseudoknot revisits the top clusters multiple times. We have revised the manuscript (Methods, p. 7, and captions of supporting figures) to clarify this point.

Minor suggestions:

*1) The term “blind structure prediction” is used many times, starting in the abstract. It would be helpful if the manuscript specified to what the predictions are blind. By reading the manuscript, I understand it is blind to prior experimental structures, but this could be made explicit.*

That is correct, the predictions are “blind” in the sense that there is no prior knowledge about structures from experiments that can be used to inform the predictions. We have made this point more explicit in the second revision (Methods, p. 5).

*2) On page 4 (of the marked up version), “sharing also sharing” should be “also sharing”.*

We thank the reviewer for noticing this error, it has been corrected in the second revision.

*3) On page 7, the manuscript states that trajectories were tested to make sure there were not “large” changes in RMSD. The manuscript should quantify “large”. Also, in the same paragraph, the hydrogen bond distance criterion is specified. Was there also a criterion for the angle of the atoms? I am accustomed to seeing a geometry requirement as well, so if one was not used, it is probably best to be clear about that*.

Trajectories were taken as having converged if the RMSD values did not change more than 3 Å from the average value after equilibration (final 500 ns of trajectory) for a duration of more than 20 ns. We did not use any criterion for the angles. We have revised the manuscript to include this information (Methods, p. 7).

*4)* *On page 9, the manuscript states “we also applied R-scape on the MSA to evaluate the conservation”. Although R-scape provides conservation information, its true value is in assessing the significance of covariations. The fact that two covariations were significant in S1 is very important support for the model. I suggest the manuscript be revised to explain this and emphasize the importance of the covariations.*

We have revised the manuscript to emphasize that the MSA is strongly supported by presence of two covarying base pairs in the results section, as suggested (Results, p. 10).

*5) On the bottom of page 9, the caveat that infernal does not handle pseudoknots is stated. I think it would be better to state this limitation up front when the manuscript states that infernal was used.*

We have revised the manuscript to mention this limitation of Infernal when the alignment is described (Results, p. 8).

*6) Figures 6, 7, 8, and 9 state that panel A is a structure with magnesium and panel B is a structure without magnesium. These must be backwards. I assume the green spheres are magnesium ions. (And starting in Figure 3, it would be helpful if the caption clarified that the green spheres are magnesium.)*

We thank the reviewer for noticing this error. We have revised the manuscript to include the suggested changes.

*7) In Figure 6, I noticed that two magnesiums intervene between S1 and S3, causing a disruption of a coaxial stack. The text comments on a base pair being disrupted in S3, but the loss of the coaxial stack seems like a more prominent change to me. It would be helpful if the text clarified whether this change occurs reliably across the two simulations.*

We originally ran only one simulation of this pseudoknot (21164-6) with Mg^2+^ ions. To address the reviewer’s question about the S1/S3 stacking, we added two more simulations with different Mg^2+^ ion starting coordinates. We found that the stacking was disrupted in the two simulations where Mg^2+^ was bound in the S1/S3 junction, whereas the stacking was preserved in the simulation where Mg^2+^ was not bound to the S1/S3 junction, suggesting that the presence of Mg^2+^ ions can indeed affect the stacking. We have revised the manuscript (Results, p. 15) to mention this observation.

*8) In Figure 9, I note that, in the absence of magnesium, the central loop is quite open in a way that seems very wrong as compared to native structures. The manuscript refers to the tertiary interactions being sparse. The magnesium-containing structure is much more compact, as expected. It would be helpful if the manuscript stated whether this difference is reliable across the repeated simulations.*

We thank the reviewer for pointing out this feature. The structures containing Mg^2+^ are indeed more compact compared to those without in the replicate simulations. We have revised the manuscript to indicate this point (Results, p. 16).

**Most significant changes to the manuscript (all in response to reviewer 4)**:

- Methods, p 5: Listed options from Infernal used in alignment.
- Methods, p 5: Explained what is meant by blind predictions.
- Methods, p 6: Explained Mg^2+^ ions were positions by hand in MOE.
- Methods, p 7: Clarified clustering method and criteria for trajectory convergence
- Results, p 8: Stated a limitation of Infernal
- Results, p 10: Clarified importance of base-pair covariance found in R-scape
- Results, p 11: Listed maximum fluctuation of bound Mg^2+^ ions
- Results, p 11, Fig S5: Added 2D RMSD analysis of replicate trajectories
- Results, p 11–12: Added analysis of base-pair persistence
- Results, p 15, Fig S12: Added discussion of effects of Mg^2+^ ions on helix stacking and results from replicates with different Mg^2+^ ion positions.
- Fig. S4: added figure showing initial Mg^2+^ ion positions.
- S2 Data: Added file showing sequences alongside predicted secondary structures.
