# Supplementary material for: Modelling the structures of frameshift-stimulatory pseudoknots from representative bat coronaviruses
Source: PLoS Comput Biol. 2023 May 19;19(5):e1011124. doi: 10.1371/journal.pcbi.1011124 (PMC10234561; doi:10.1371/journal.pcbi.1011124)
Supplement: Attachment — Submitted filename: Sekar_etal_Response to reviewer comments.doc [file pcbi.1011124.s025.doc]

**Response to reviewer comments**

**Reviewer #1**

*In this study, the authors provide representative bat-CoVs frameshift-stimulatory pseudoknot structures that are distinct from SARS-like CoVs and study their key conformational features shedding light on frameshifting mechanisms and anti-CoV drugs. Two beta-CoV sequences and two alpha-CoV sequences are selected after clustering based on genetic distances. Pseudoknot structures are obtained from computational predictions of 2D and 3D structures in combination with microsecond molecular dynamics simulations. Although the four selected representative pseudoknots have different numbers of stems, all of them are predicted to adopt two folding topologies: 5’ end threaded through a ring at stem junction and unthreaded along the outside of the ring, same as SARS-CoV-2 frameshift pseudoknot. Stem 1 in the representative pseudoknots studied here has high similarities in both length and sequence identity, and S1 conformations also match those in SARS-CoV-2 frameshift pseudoknot. Based on these findings, the authors suggest the possibility to find broad-spectrum anti-CoV therapeutics by inhibiting -1 programmed ribosomal frameshifting.*

*The study is well-designed - from sequence to structure and function. The predicted structures are carefully selected - using consensus lowest-energy 2D structures and excluding disfavored 3D structures. The findings in this work support previous discoveries from SARS-CoV-2 pseudoknot in the 5’-end threaded ring-knot conformation and generalize the insights to a wide range of CoV pseudoknots. However, some of the unique findings in this work can be strengthened, such as different RNA foldings introduced by variations in sequence.*

We thank the reviewer for these positive comments.

*Major points:*

*The authors performed clustering on 48 bat-CoV sequences and selected representative sequences in this study. However, how sequence affects the RNA conformation is not clearly stated. The beta-CoV pseudoknots adopt a three-stem architecture while alpha-CoV pseudoknots don’t. Is this a coincidence?*

The fact that the bat beta-CoVs form 3-stem structures is not particularly surprising, as previous work on sequence conservation in all beta-CoVs (of which the bat beta-CoVs are only a subset) suggested that a 3-stem architecture is common to them (Rangan *et al*. (2020) *RNA* 26:937). However, to our knowledge no such study of alpha-CoVs has been done, so we do not know *a priori* if 3-stem pseudoknots are uncommon or absent in all alpha-CoVs. That said, while responding to comments from other reviewers, we redid the sequence alignment using an expanded set of bat-CoV sequences (up from 48 sequences listed under taxID 1508220 [bat CoVs] in NCBI Virus having full coverage of the frameshift signal to a new total of 63, thanks to sequences that had been newly listed since we originally did the alignment). These additional sequences, in concert with an improved alignment, revealed some extra clusters of bat alpha-CoVs, some of which also adopted a 3-stem architecture. Hence the most noticeable difference between alpha- and beta-CoV pseudoknots was that alpha-CoVs featured 2- and 4-stem pseudoknots *in addition* to 3-stem pseudoknots, not instead of them.

We have revised the manuscript to clarify this point in the Discussion section (p. 21).

*Does any of the sequences favor a long and stable pseudoknot Stem 2?*

To assess whether sequences others than those we modelled structurally might favor a longer stem 2, we predicted the secondary structure of all 63 sequences in our alignment (along with SARS-CoV-2 as the representative of the SARS-like cluster of CoVs), using pKiss, hotKnots, PKNOT, and NUPACK. As seen in Table S1 in the revised manuscript, the pseudoknot base-pairing is predicted to be close to identical (in some cases exactly so) within each cluster, with S2 varying in length from 3–8 base-pairs.

We have revised the manuscript to compare the lengths of stems 1 and 2 (Discussion, p. 22), and added Table S1 to the Supplementary information.

*A longer L3 is reported in several pseudoknots in this work compared with SARS-CoV-2. Is this due to sequence variations?*

Yes, that appears to be the case. We note that the experimentally reported length of L3 in the SARS-CoV-2 pseudoknot varies in the literature from 2–5 nt (Refs. 18, 21–23, 48 in the revised manuscript). More generally, the length of the “loop” segment between S1 and S2 seems to be the main feature driving gross differences in structure between the pseudoknots: in many cases it’s large enough that one or even two separate stems can form within this segment, in others it’s too small to allow any extra stems to form. We have clarified this point in the revised manuscript (Discussion, p. 22).

*Minor points:*

*1. I was wondering if the authors tested and examined the Mg2+ binding sites or the convergence of MD trajectories regarding the initial Mg2+ ions placement. The results that the presence of Mg2+ increased the fluctuations of L3 in the two unthreaded beta-CoV pseudoknots and disrupted some base pairs in the two unthreaded alpha-CoV pseudoknots are very interesting.*

The structural effects of Mg^2+^ ions are difficult to ascertain even from high-resolution experimental models: no ions are seen in either of the two cryo-EM structures of the SARS-CoV-2 pseudoknot, and different numbers of ions are seen in the two crystal structures (12 ions in 7LYJ and 9 in 7MKY), not necessarily in the same positions. Given the complete lack of structural information about the bat-CoV pseudoknots we study in our manuscript, our purpose in comparing the simulations with and without Mg^2+^ was not to provide a definitive measure of the effect of Mg^2+^ on the structures, but rather to get a qualitative sense of whether the ions might induce large structural changes, as can occur in some RNAs. Our results suggest that they don’t—the structures with and without Mg^2+^ are broadly similar.

That said, we recognize that it would be helpful to explore more thoroughly the reliability of the simulations with Mg^2+^ by testing whether simulations that have different initial ion placements converge (Fig. R1). To do so, we focused on the KU182958 pseudoknot (GLGC2 in the new nomenclature), testing 3 different initial ion placements in the threaded conformer (half of the bat-CoV pseudoknots have only a threaded conformer; we presume that for the others, the threaded conformer is the dominant structure as it is for SARS-CoV-2 (Ref. 26 in the revised manuscript). Looking at the top 3 clusters from each of the simulations, we found that even though some Mg^2+^ ions were found in the same position when comparing different clusters within a given trajectory, suggesting that they were relatively stable, the coordinates of these stable ions were not the same when comparing between the trajectories starting with different initial ion placements, suggesting that there are multiple sites to which Mg^2+^ may converge but that 1 μs is not sufficient to identify them all and converge on the most stable positions. However, the top 3 clusters from all 3 simulations did yield structures that were identical in terms of the base-pairing and very similar in terms of the 3D structure, differing mostly in the flexible loops, confirming our conclusion that Mg^2+^ did not induce any significant changes in structure. We also did a similar analysis of the effects of initial Mg^2+^ ion positions for the Anlong-172 pseudoknot, finding similar results. We have revised the manuscript to include these new results (Results, p. 13, and Fig. S7).

Regarding the effects of Mg^2+^ on fluctuations, we found that the different trajectories starting with different ion positions described above showed noticeable variability in the RMSF values for L3. In fact, the differences between the L3 fluctuations that arose for the same pseudoknot because of different ion placements were similar in size to the differences in flexibility we had noted when comparing simulations with and without Mg^2+^. We therefore cannot be confident that the effects of Mg^2+^ on fluctuations we had noted in the manuscript are reliable. We have revised the manuscript to remove all comments comparing the fluctuations in simulations with and without Mg^2+^.

**Fig. R1**: Top clusters from MD simulations of GLGC2 pseudoknot with 3 different ion starting positions.

*2. The authors reported alternative 2D structures of KF294282, and some 3-stem 2D structures were not stable in MD simulations. Did these MD trajectories (starting from different predicted models of this pseudoknot) converge to the same 2-stem 2D structure?*

We agree with the reviewer that it would be interesting to see if the trajectories starting from the 3-stem structures eventually converged to the same 2-stem structure. However, convergence for such large-scale structural reorganizations often takes a very long time and is thus overly costly computationally. Given our finite computational resources, we were unable to test this proposition by continuing the simulations long enough to see convergence from the unstable 3-stem structure to the stable 2-stem structure.

**Reviewer #2**

*In this manuscript, the authors build microsecond molecular dynamics simulations for frameshifting elements (FSEs) of 4 bat coronaviruses. Different initial 3D models are used, and simulations with and without Mg^2+^ are both performed. The two beta-CoVs display similar 3-stem H-type pseudoknots as SARS-CoV-2, while the two alpha-CoVs have a 4-stem and a 2-stem pseudoknot structure. Despite this difference, all FSEs adopt both 5’ end threading and non-threading topologies, suggesting the FSE structural heterogeneity of bat-CoVs.*

*Despite the potential utility for future drug targeting against bat-CoVs, more explanations are needed for the Methods section, and deeper analysis can be added to the Results and Discussion sections to contribute more therapeutic insights. Overall, the manuscript requires major revisions to be considered for publication. Below are the detailed comments:*

We thank the reviewer for the feedback and comments.

*1. In Methods, subsection “Bat-CoV pseudoknot sequences”, the authors perform clustering for 48 bat-CoV sequences. How exactly are the “pairwise genetic distances” calculated and clustered? Do the 5 clusters correspond to different subgenera? Moreover, in Fig.1, only 25 sequences are displayed. What happen to the other sequences, do they form different clusters?*

We regret the confusion that was caused by our presentation of the sequence alignment and clustering. As mentioned in the main text, the sequence alignment and clustering had been published previously in Ref. 17 and was not an original part of the present work. To avoid giving the impression of double publication of this component of the work, we had decided to provide fewer details about the method (since it is described elsewhere) and to display a more compact summary of the results from the earlier work in Fig. 1 by deleting many of the sequences (primarily those that were particularly close genetically). This reasoning explains why Fig.1 shows only a subset of all the sequences used in clustering: it is intended to provide the context for the sequences we study in the current work without appearing to duplicate the previous work. We had expected readers who want more detail about the previous work to consult Ref. 17.

However, prompted by comments below from Reviewer 4, we have re-analyzed the sequence alignment and clustering using new tools suggested by the reviewer, and we have expanded the number of sequences included in the analysis by including entries that have been added to NCBI Virus since our original alignment was done. Since this new clustering analysis is now part of the original work presented in this manuscript, a full description of the methods is required. We have therefore revised the manuscript to describe the new sequence alignment and clustering analysis in detail (Methods, p. 5, Results p. 7–9). We have also expanded Fig. 1 to include all the sequences in the full phylogenetic tree, and we have shown the alignment in new supporting material (Supplementary File S1).

*2. In Methods, subsection “Blind structure prediction”, can the authors briefly explain why they select HotKnots and pKiss for 2D structure prediction and FARFAR2 for 3D prediction, and why these programs are enough?*

For 2D structure prediction, we selected pKiss and HotKnots as they are well-established algorithms that have been used relatively widely. In response to similar comments from reviewer 4 regarding the choice of 2D structure predictors, we also added in the predictions from two more prediction tools, PKNOT and NUPACK, to help make the consensus prediction more robust. One or both of the new algorithms agreed with the previously predicted consensus of the most stable pseudoknot structure in half the cases; in the rest, neither of them predicted any pseudoknot. The revised manuscript now lists which algorithms were involved in the consensus prediction in Table S1. We note that the new sequence alignment and consensus 2D prediction changed the predicted base-pairing for KF294282 (Neixiang-64 in the new naming system) to the second of the 3-stem structures that we had tried simulating for this pseudoknot.

For 3D structure predictions, we found in our previous work on the SARS-CoV-2 pseudoknot (ref. 20 in the revised manuscript) that FARFAR2 yielded the most consistently reliable predictions, in terms of tending to be stable in MD simulations and reasonably close to the subsequently published experimental structures. Some other predictors performed reasonably well, too, but their output was sufficiently similar to the results from FARFAR2 that we judged it unnecessarily duplicative to perform multiple predictions and simulations (especially given that there are many times the number of pseudoknots to model here as in our previous work on the SARS-CoV-2 pseudoknot, requiring more efficient use of computational resources).

We have revised the manuscript to describe and motivate the choice of these prediction methods more clearly (Methods, p. 5 and Results, p. 9–10).

*3. In Methods, subsection “Molecular dynamics simulations”, can the authors give more details on how they place the Mg^2+^ ions, and is there any rational behind? Placing Mg^2+^ can be tricky as their placement could bias the simulation.*

We agree with the reviewer that the choice of initial coordinates for the ions is a non-trivial question that may bias the simulation. However, as there is no information available about what role, if any, Mg^2+^ plays in bat-CoV pseudoknot structure, nor how many ions are present and where they are located, our only point of reference is the SARS-CoV-2 pseudoknot (the only CoV pseudoknot whose structure has been solved at high resolution). Even that information is not particularly helpful, however—although the available crystal structures (refs. 22 and 23 in the revised manuscript) do show Mg^2+^ ions located along S1, S3, and in threading region and junction between S1 and S3, they do not agree either on the number of the ions nor their position (some ions have similar positions in the two structural models, but others do not). Moreover, both of these crystal structures show linear conformations that are inconsistent with published cryo-EM results (refs 18 and 21 in the revised manuscript) and unpublished SAXS data from our collaborator Lois Pollack (Cornell Univ.), all of which show a prominent bend at the junction with S3, suggesting that they may be affected by significant artifacts arising from crystal contacts. Hence it is unclear to what extent the number and position of the Mg^2+^ ions seen in the crystal structures is valid and/or relevant.

In our models, we placed Mg^2+^ manually near the backbone at locations guided by (although not identical to) the crystal structures for the SARS-CoV-2 pseudoknot: along S1, in the threading region, and in the junctions between stems. Importantly, as we noted above in response to reviewer 1 (p. 2–3), the goal of the simulations with Mg^2+^ was to provide a qualitative sense of whether Mg^2+^ ions caused large, obvious changes to the predicted structures. The result we found was that they did not, for any of the pseudoknots. Nevertheless, the fine details of the structural models we produced in the presence of Mg^2+^ could well be biased by choices made in placing the ions, and the results with Mg^2+^ should therefore be considered suggestive more than anything else. To explore how much the results with Mg^2+^ change when the ion placement is changed, we redid the simulations of the KU182958 pseudoknot (GLGC2 in the new naming system), as well as one of the new pseudoknots (Anlong-172), using different initial ion positions. Importantly, the results (see Fig. R1 above and Fig. S7) show that changing the initial ion placement does not change the base-pairing or significantly alter the overall 3D structure, but it does affect the flexible regions and influence the location of the ions. These results suggest that the Mg^2+^ positions are likely not truly equilibrated in our 1-μs simulations, which is not surprising given the large energy barriers that can be involved in moving Mg^2+^ ions that make specific local contacts with RNA.

We have revised the manuscript to explain the limitations of the simulations with Mg^2+^ more clearly, and to present the investigation of Mg^2+^ position variability (Results, p. 13 and Fig. S7).

*4. In the first paragraph of Results, the authors choose to model the 100-nt FSE sequences “starting 6 nt downstream of the UUUAAAC slippery sequence”. Why not include the slippery site and the spacer region? Also, “an additional 3-4 nt were added to the 5’ end of each RNA” after the 2D structure predictions. Why not add these nucleotides during 2D predictions?*

During frameshifting, the ribosome is stalled on the slippery sequence before reading through the pseudoknot, and the 6-nt spacer sequence is inside the RNA entry pore of the ribosome. Because these nucleotides are sequestered inside the volume of the ribosome, it is physically impossible for them to be part of the structure that stimulates frameshifting. They might take part in alternative structures that form in the absence of the ribosome, but our focus in this manuscript is on structures that stimulate frameshifting, hence we ignore those other possibilities. These considerations motivate our decision not to include the slippery sequence and spacer sequence when building secondary structure models. Nevertheless, when building 3D predictions, we need to include part of the spacer sequence (which is forced to remain single-stranded because it is not included in the 2D predictions) because these extra nucleotides are required to distinguish between threaded and unthreaded structures, as found in earlier work on the SARS-CoV and SARS-CoV-2 pseudoknot (Park *et al.* (2011) *JACS* 133:10094, which did not add in the extra nucleotides and as a result failed to identify the existence of different fold topologies, and Ref. 20 in the revised manuscript, which did).

We have revised the manuscript (Results, p. 9–10) to explain the motivation for these choices more clearly.

*5. In the second paragraph of Results, the authors “selected one example from each group as the initial structures for MD simulations”. How exactly are these initial structures selected?*

FARFAR2 provides the structure of 10 lowest energy cluster centers. From each group we picked the one that had the lowest energy reported by FARFAR2. We appreciate the concern implicit in this comment—which was raised more explicitly by reviewers 3 and 4—that this choice might bias the results of the MD simulations. To address this concern, we added simulations starting from the next two lowest-energy structures predicted by FARFAR2 for the threaded conformations of each pseudoknot. We focused on the threaded conformation because (i) half of the pseudoknots have only a threaded conformer and (ii) it is the dominant one in all experimental work on the structure and dynamics of the SARS-CoV-2 pseudoknot (refs 18, 21–23, 26 in the revised manuscript). We found that the structures obtained after MD simulations starting from different initial predictions were all very similar qualitatively (in terms of the base-pairing, arrangements of helices, and even overall density of tertiary contacts, but not the details of the tertiary contacts nor the orientations of the loops or flexible stems like S3 or S4), providing confidence that the results are not an artifact of the choice of initial prediction. See Fig. R2 below for the comparison done for the GLGC2 pseudoknot (previously called KU182958).

We have revised the manuscript (Methods, p. 6; Results, p. 11) to describe how we selected the examples for use in MD simulations, and added in the results from the new simulations starting from different initial structures in Figs. S5 and S9–14.

**Fig. R2**: Top clusters from MD simulations starting from different 3D predictions for GLGC2 pseudoknot.

*6. During the 3D structure prediction and MD simulation, has the 2D structure folding changed? The authors can track the base pairing of different stems to help show the stabilization/convergence of their MD models.*

This analysis was implicit in the figures that were included in the manuscript, but not discussed much explicitly in the text. Fig. S1 in the original manuscript showed the base-pairing from 2D secondary structure predictions, and Figs. 3–6 showed the base-pairing from the MD simulations. We did not show the base-pairing for the 3D predictions from FARFAR2 because these predictions conserved the base-pairings that were used as an input.

We have revised the Results section of the manuscript to mention that the base-pairing does not change from 2D prediction to 3D prediction and to discuss for each pseudoknot the changes (if any) seen at the MD simulation stage.

*7. The authors observe that non-threaded FSE structures have higher RMSF in the Loop 3 region when Mg^2+^ ions are present. Why is this the case, shouldn’t Mg^2+^ helps stabilizing the structure? Does this suggest the placement of Mg^2+^ can be improved?*

The effect of Mg^2+^ on L3 fluctuations was noted in the original manuscript for the threaded and unthreaded conformers of KU182958, and for the threaded conformer of LC469308. Considering first the unthreaded conformer of KU182958, it turned out that the fluctuations in L3 were lower in the absence of Mg^2+^ because the unthreaded 5′ end interacted sufficiently with L3 so as to reduce its fluctuations; in contrast, the 5′ end interacted less with L3 when Mg^2+^ was present and instead showed transient steric clashes with it, increasing the fluctuations. For the threaded KU182958 conformer, one of the Mg^2+^ ions moved between different positions within S3 when comparing the top 3 clusters from this trajectory, which led to enhanced fluctuations in the L3-S3 region. For the threaded LC469308, Mg^2+^ ions moved within S3 and L3 when comparing the top 3 clusters from this trajectory, which again led to enhanced fluctuations.

That said, however, please note that as mentioned in response to reviewer 1 above on p. 3, we are removing discussion of the effects of Mg^2+^ on fluctuations because the simulations done using different ion positions suggests that we cannot draw reliable conclusions about the effects of Mg^2+^ without more knowledge about Mg^2+^ ions in these pseudoknots or much more extensive simulations of their effects, both of which are beyond the scope of this work.

*8. The authors mention several times about the bulges in stems. What is the significance of this?*

Earlier work on the SARS-CoV pseudoknot showed that mutations to the bulges in S2 and S3 had significant effects on frameshifting (ref 44 in the revised manuscript), suggesting they played an important role. We have revised the manuscript to clarify the reason for interest in the bulges (Results, p. 12).

*9. The authors mention the SARS-CoV-2 FSE structure many times in the manuscript. A supplementary figure can be provided for this structure, highlighting features like the threaded ring conformation.*

We have revised the manuscript to include a figure showing published structural models of the SARS-CoV-2 pseudoknot in Fig. S4.

*10. In Discussion, the authors discuss the impact of S3 on stimulating -1 PRF. Exact frameshifting efficiency percentages can be provided for different viruses.*

Please note that frameshifting efficiency values are almost always measured experimentally using artificial reporter constructs, rather than measuring viral transcript levels, and the results depend strongly on the experimental conditions of the assay. For example, measuring the same dual-luciferase reporter construct in two different cells can yield values that differ by as much as a factor of ~2 (as concrete examples, consider the results for SARS-CoV from refs 44 and 48, where yeast, HEK, and HELA cells produced numbers for the same frameshift signal ranging from ~15% to ~30%); these number may in turn differ from the values obtained in cell-free assays. The “exact” frameshifting efficiency values are therefore hard to pin down—the values obtained from reporter systems are only a condition-dependent estimate. Meaningful comparisons between frameshifting levels can only be done when all measurements have been done in the same way. We kept the discussion qualitative precisely to avoid the problems inherent with trying to discuss “exact” frameshifting values.

That said, we appreciate the intent of the reviewer’s comment, and we have revised the manuscript (Discussion, p. 22) to specify the frameshifting levels observed along with the measurement conditions used.

*11. In Discussion, the authors suggest that “S4 may reduce -1 PRF by interfering sterically with contacts between the pseudoknot and ribosome, or with the ability of S3 to bend with respect to S1/S2”. Have the authors checked the dominant motions of different bat-CoV FSE trajectories? Does KY770854 trajectory show lack of S3 bending compared to the others?*

We thank the reviewer for the suggestion. We investigated the degree to which S3 bends relative to S1 in the simulations. We defined the S1 axis via a line through the average positions of the two bp at each end of the helix, then calculated the angle of this line with respect to the line from the end of the S1 helix to the end of the S3 helix. We then compared the average S3 bend angle for different pseudoknots to see if the trajectory for the pseudoknot with anomalously low frameshifting efficiency was any different (Fig. R3). In fact, we did not see any noticeable decrease for KY770854 (now known as Anlong-44).

**Fig. R3**: Standard deviation of the S1-S3 bend angle.

However, we note that this analysis is not particularly definitive, as it looks at the flexibility of S3 with respect to spontaneous random fluctuations, which is not a direct test of our proposal that S3 might resist displacement by the ribosome. To test the proposal properly, we would need to include the force from the ribosome acting on S3, which are currently unknown in terms of the location at which force is applied, as well as the direction and magnitude of the force. The lack of correlation between S3 bending flexibility in the isolated pseudoknot and frameshifting efficiency therefore doesn’t disprove the proposal, it just doesn’t support it.

We have revised the proposed explanation to note this lack of support from the analysis of S3 bending flexibility (Discussion, p. 22, and Fig. S15).

*12. In Discussion, the authors state “these structural similarities between pseudoknots representative of the different varieties found in bat CoVs suggest that it should be possible to find small-molecule ligands that can inhibit -1 PRF in a broad spectrum of CoVs”. Can the authors suggest specific FSE target regions based on the structural similarities?*

We did indeed suggest a general region that could be so targeted: in Fig. 7, we showed that the region including the base of S1 and the ring around the threaded end is similar in all the structures, and hence could be targeted by ligands that might be able to bind all the pseudoknots. However, going further than this analysis (for example, by identifying and validating specific docking sites in the proposed region of interest) is beyond the scope of this manuscript and best addressed in future work.

*13. In Discussion, the authors cite that some PRF inhibitors demonstrate different effects for beta-CoVs and alpha-CoVs, while some have similar effects for both. Can the authors elaborate more on this, by checking the binding pockets of the different inhibitors and relating with the structural differences between beta and alpha CoVs?*

We agree that it would be interesting to explore the reason why some PRF inhibitors appear to be somewhat selective between CoV families whereas others are less so. However, addressing this question involves exploring the mechanism of action of the inhibitors, which in most cases has not been reliably established. Indeed, binding of PRF inhibitors to bat-CoV pseudoknots has not been characterized experimentally either for binding affinity or for binding location. This question is therefore beyond the scope of our manuscript, and best addressed in future work that might be able to combine experimental studies of inhibitor binding with structure models of binding pockets.

*14. Throughout the manuscript, the authors use accession numbers instead of virus names. I often need to check backwards to see which accession number is for which virus, and is it beta or alpha coronavirus. The authors should improve this by giving the virus names as well.*

We appreciate the reviewer’s point that accession numbers are not the easiest ‘name’ to remember for viruses. Most of the viruses included in our work do not seem to have a formal name (the organism name listed in NCBI Virus is simply “bat coronavirus”), which is what motivated our choice to use accession numbers as identifiers. However, NCBI Virus does list isolate names for each of the viruses we modeled. We have therefore revised the manuscript to use these isolate names as the primary identifiers in the manuscript for those viruses that we modelled structurally. However, we kept the accession numbers as the primary identifiers in the figures and/or tables involving alignments, sequence clustering, etc…, because the format of accession numbers is more consistent than the format of isolate names.

The correspondences between accession numbers and names for the viruses that we modeled structurally in the revised manuscript are:

- KU182958 (cluster 1, unchanged from original manuscript): bat CoV isolate GLGC2 (abbreviated to GLGC2)
- LC469308 (cluster 2, unchanged from original manuscript): bat CoV isolate Vs-CoV-1 (abbreviated to Vs-CoV-1)
- KY770854 (cluster 3, unchanged from original manuscript): bat CoV isolate Anlong-44 (abbreviated to Anlong-44)
- MN535732 (cluster 4, new cluster): bat CoV isolate BtCoV/21164-6-alt/M.dau/DK/2015 (abbreviated for convenience to “21164-6”)
- KF294269 (cluster 5, new cluster): bat CoV isolate Anlong-8 (abbreviated to Anlong-8)
- MG916902 (cluster 6, new cluster): bat CoV isolate BtCoV/Rh/YN2012_Rs4125 (abbreviated for convenience to “Rs4125”)
- KF294276 (cluster 7, new cluster): bat CoV isolate Anlong-172 (abbreviated to Anlong-172)
- KF294282 (cluster 8, formerly called cluster 4): bat CoV isolate Neixiang-64 (abbreviated to Neixiang-64)

*15. For all 3D structure plots, the authors should label the 5’ and 3’ ends, as well as the stems.*

We have added these labels to the figures as requested (Fig. 2–10).

**Reviewer #3**

*In their manuscript "Modelling the structures of frameshift-stimulatory pseudoknots from representative bat coronaviruses", Sekar et al. employed established RNA structure prediction algorithms and subsequent all-atom MD simulations to model the structures of RNA pseudoknots crucial for the proliferation of coronaviruses. Knowledge of these structures might have important implications for finding antiviral drugs.*

*The manuscript tackles an important question and is very clearly written. I enjoyed reading it!*

We thank the reviewer for the positive comments.

*The authors miss several opportunities to validate their methods and results, which would strengthen their conclusions.*

*First, the SARS-CoV-2 pseudoknot was not considered because a wealth of structural information already exists. However, this structural information would render this pseudoknot a good candidate to test the structure prediction protocol by comparing the results with the available experimental data.*

All but the first step of the structure prediction protocol (*i.e*., everything but base-pairing predictions) has already been applied to the SARS-CoV-2 pseudoknot, in Ref. 20 of the revised manuscript. The predictions for the SARS-CoV-2 pseudoknot were found to be reasonable when compared to subsequent experimental results: the prediction of an unusual 5′-threaded conformer was confirmed by subsequent structural studies, the “bent” structure of the 5′-threaded conformer was similar to what was seen in cryo-EM (refs. 18 and 21), the arrangement of S1 and S2 was similar to what was seen in crystal structures (Refs. 22 and 23), and the prediction of multiple conformers with different threading topologies was confirmed by single-molecule folding studies (Ref. 26). These results give us confidence that the general approach is leading to a reasonable approximation of the actual structures formed by CoV pseudoknots.

That said, however, we did not include the base-pairing prediction in previous work, as we had previous experimental data on base-pairing from studies of the SARS-CoV pseudoknot. To make a more complete comparison with the current protocol, we therefore re-ran the structure prediction starting with base-pairing prediction as done in this manuscript, to test if it matched experimental data. We found that the consensus 2D structure prediction approach yielded the same base-pairing as found in the cryo-EM structures (this prediction is now listed in Table S1 in the revised manuscript).

We note, however, that a rigorous comparison of computational and experimental models of the SARS-CoV-2 pseudoknot structure is complicated by the fact that the five published experimental structures all disagree on various features, including aspects like base-pairing and tertiary interactions. Some of these differences can be seen below in Fig. R4 showing 6 structural models from cryo-EM (2), crystallography (3), and computation (1). Given the variability in the experimental results, a qualitative comparison such as we described above is the best that we can do; a more quantitative analysis would require extensive work that goes well beyond the scope of our current paper and would best be the subject of its own paper.

**Fig. R4**: Published structural models of SARS-CoV-2 pseudoknot: (A) cryo-EM, (B) crystallography, (C) simulation. (D) Base-pairing in experimental models (asterisks indicate disagreements between models).

We have revised the manuscript to include the comparison of experimental and computational structural models for SARS-CoV-2 in Fig. S4, and to include the predicted 2D structure for SARS-CoV-2 in Table S1 (noting that it is the same as in the cryo-EM structures).

*Second, for each considered pseudoknot and ion concentration, only one simulation was carried out and the simulations were considered equlibrated after 500 ns. How was it determined if a simulation is in equilibrium? If the system is equilibrated, multiple simulations of the same length would give the same results and support the notion that equilibrium is reached. Replicates of simulations are standard in the field to estimate the statistical errors and to prevent that the reported results are merely anecdotal. Therefore, at minimum, the authors should provide replicates for one of the pseudoknots.*

To determine if the simulation had equilibrated, we monitored the RMSD values to look for large jumps. If no such changes were observed, we concluded that system was in equilibrium. However, we appreciate the reviewer’s concern and therefore performed 3 simulation replicates for two of the structures (threaded structures for the pseudoknots from the beta-CoVs KU182958 and LC469308, now respectively renamed GLGC2 and Vs-CoV-1, in both cases without Mg^2+^) to verify that the results were the same. Comparing the top clusters from each of the replicates for the two pseudoknots studied this way (Fig. R5) shows that the qualitative features of the structures (base-pairing and arrangements of helices but not detailed tertiary contacts or orientations of loops and flexible stems like S3) were the same and confirms that the system is equilibrated. However, we note that the work we did in response to reviewers 1 and 2 above—looking at replicates done in the presence of Mg^2+^ ions placed at different initial locations—suggests that the ion positions may not be fully equilibrated. Equilibration is also confirmed by the fact that the top clusters tend to recur multiple times in the trajectories (see response to point 9 of reviewer 4 below).

We have revised the manuscript to include these new results confirming that, at least in the absence of Mg^2+^, the simulation length is sufficient for equilibration of the RNA structure (Results, p. 13, and Fig. S6).

**Fig. R5**: Secondary structure, tertiary contacts, and 3D structures for top clusters from 3 replicates starting from the same initial 3D prediction for (A) GLGC2 and (B) Vs-CoV-1 pseudoknots.

*Third, the authors write that within one of the categories, the structural differences between predictions were minor and therefore only one structure was chosen for MD simulations. What is the criterium for deciding that one starting structure suffices? Are the conformation of the other predictions seen in the simulation started from the one predicted structure? Do simulations started from different predicted structures give rise to the same structural ensemble as would be expected for equilibrated simulations?*

We had picked the lowest-energy predicted structure from FARFAR2 for each of the threaded and unthreaded conformers as the initial structure for simulations. These structures were usually visually similar to many of the other FARFAR2 predictions, differing primarily in the flexible regions of the structure, but we did not apply any quantitative criterion.

In order to make sure that the choice of initial structure does not overtly bias the outcome of the simulation, we did additional simulations starting from the next two lowest-energy structures predicted by FARFAR2, for every pseudoknot cluster for which such alternative predictions were available (in some cases, there were fewer than 3 topologically acceptable structures were predicted, in which case we did replicates of acceptable structures). We focused on the threaded conformers only, because we did not have sufficient computational resources to do the unthreaded conformers as well and (as noted above) the threaded conformers are expected to be the dominant ones in frameshifting. We found that the results from the replicates with different initial structures did indeed look very similar to the original results, with all helices the same and the only notable differences seen in the flexible loops or the orientation of stem 3 (which is also expected to be flexible, given that it is connected to stems 1 and 2 by flexible loops and it has differing orientations in each of the published experimental structures for the SARS-CoV-2 pseudoknot). Selected examples are shown in Fig. R6.

We revised the manuscript to present these additional simulations showing the results obtained when starting from other initial 3D structure predictions (Fig. S5, S9–14).

**Fig. R6**: Results for top clusters from 3 replicates starting from the three lowest-energy 3D predictions for (A) GLGC2 and (B) Anlong-172 pseudoknots.

*Typo on P. 19: "a high level sequence of sequence identity"*

We have corrected this error.

**Reviewer #4**

*In “Modelling the structures of frameshift-stimulatory pseudoknots from representative bat coronaviruses,” Sekar et al. build all-atom models of four diverse bat coronavirus frameshift elements using RNA secondary structure prediction, 3D modeling with FARFAR, and molecular dynamics with AMBER. The four bat models are compared with each other and also compared to the experimentally-determined structure for SARS-CoV-2. Overall, the modeling is interesting as a way to generate hypotheses for the structures, but the hypotheses are untested.*

We agree that the models we present are just that—models, as yet untested experimentally. However, given that there is still no high-resolution structure for any alpha- or beta-CoV pseudoknot other than SARS-CoV-2, we feel that computational models are a good starting point in the absence of any other structural information about bat-CoV pseudoknots.

*Major Revisions: [numbering added for convenience]*

*[1.] The modeling software tools used are state-of-the-art, but the modeling effort did not take advantage of the evolutionary conservation of structure (i.e. the underlying homology of the sequences). The manuscript does compare the models, but it is not made clear if the differences are a result of the evolution of structure or whether the modeling effort failed on some level because the expected conservation was not used. For example, secondary structure prediction was performed using single sequences, which is known to be helpful but not perfectly accurate. The best practice would have been to test the secondary structure models using a sequence alignment across the bat coronavirus frameshift elements. For KF294282, a structure different from the other 3 was predicted, but the manuscript is not clear whether the sequence is capable of folding in the same structure. An example of an alignment for conservation analyses are found in related literature [JACS. 2021. 143:11404]. Covariations of base pairs should have been tested for significance [Nature Methods. 2017. 14:45]. In the absence of comparative analysis, I am skeptical of the quality of the secondary structure models.*

We thank the reviewer for the suggestion to make the alignment more robust by using additional tools, as it has led to some significant changes to our analysis of the subset of bat-CoV pseudoknots originating from alpha-CoVs. We note that a conservation analysis has already been done for the beta-CoV family by Rhiju Das’ group (Rangan *et al*. (2020) *RNA* 26:937), finding that the 3-stem architecture is conserved across the beta-CoVs. Our base-pairing (2D) predictions for two beta-CoV pseudoknots are consistent with this previous work. However, such an analysis has not, to our knowledge, been published for alpha-CoVs. The reviewer’s suggestion that we should include a comparative sequence analysis is therefore particularly appropriate for the alpha-CoV work, especially since the alpha-CoVs appear to support different base-pairing architectures.

In response to the reviewer’s comments, we revisited the multiple sequence alignment and phylogenetic tree construction along the lines suggested by the reviewer. For this analysis, we used additional bat-CoV sequences (taxID 1508220 in NCBI Virus) that had been added to the database since we did the original alignment, bringing the total number of bat-CoV sequences in the alignment (i.e., those with full coverage of the frameshift signal) to 63. First, a covariance model was constructed and calibrated in Infernal (Ref. 32 in the revised manuscript), using the SARS-CoV-2 frameshift signal (starting from the slippery sequence) and the SHAPE probing data provided in Ref. 27 of the revised manuscript (Schlick *et al*. (2021) *JACS*). This covariance model was used to search for regions homologous to the SARS-CoV-2 frameshift signal in the bat-CoV sequences being considered and thereby generate a multiple sequence alignment. The resulting alignment was then used to build a new phylogenetic tree based on inter-sequence distances (calculated in Geneious Prime).

We further tested the reliability of this alignment in two ways: (1) First, we used R-scape (ref. 43 in the revised manuscript), as suggested by the reviewer, to test how much of the secondary structure of the SARS-CoV-2 frameshift signal is compatible with each of the sequences in the alignment, taking into account the possibility of co-varying base-pairs. The results (Fig. R7 below) suggest that stem 1 is highly conserved across the alignment, but stem 3 is much less so (note that because pseudoknotted pairs are not captured in the analysis, there are no results for stem 2). These results are consistent with the individual secondary structure predictions for the selected representative sequences for each cluster, given that S1 is similar across all bat CoV pseudoknots, but S3 is consistent only among beta-CoV pseudoknots and it is much more variable among the alpha-CoV pseudoknots (including being absent in some). (2) Second, we used the same four 2D structure predictors that we had applied to the 8 representative sequences studied in detail in our revised manuscript to predict—individually—the secondary structures of all sequences in the alignment. The results of this analysis are shown in Table S1 in the revised manuscript. We found that the predicted secondary structures were consistent for the sequences within a given cluster, with only a few exceptions, supporting the identification of the clusters from the alignment and providing yet more evidence that the differences we found between beta- and alpha-CoV pseudoknots are not simply a failure of the prediction models for the particular sequences we chose to study.

We have revised the manuscript to describe these new analyses and their results. The sequence alignment is shown in Supplementary File S1, the full phylogenetic tree in Fig. 1, the R-scape analysis in Fig. S1, and all the 2D structure predictions in Table S1.

**Fig. R7**: R-scape analysis of conser-vation of SARS-CoV-2 pseudoknot structure within bat-CoV pseudoknot alignment.

Regarding the structure of the KF294282 pseudoknot (now renamed Neixiang-64), we did, in fact, initially predict base-pairing that was similar to the 3-stem architecture found in beta-CoVs, as we discussed on p. 11–12 of the original manuscript. However, as discussed there, the 3^rd^ stem was not stable in MD simulations, nor was it stable for simulations of an alternate 3-stem pairing, implying that this pseudoknot has only 2 stable stems. Interestingly, the 3-stem prediction was an outlier among the other two sequences in the same cluster, where the structure was predicted to contain only 2 stems.

*[2.] Another aspect of concern is that many analyses are qualitative. An example of this is the comparison of molecular dynamics simulations. Single simulations were run for each 3D model with or without magnesium. The comparisons lack necessary controls because there is no estimation of the variability for simulations of the same coordinates. I expect to see multiple simulations for starting coordinates and solvent conditions if simulations are to be compared.*

We respectfully emphasize that the purpose of the manuscript is primarily qualitative, ***not*** quantitative, which is why we focused on qualitative aspects of the models. The aims are to determine if bat-CoVs pseudoknots commonly have the unusual 5′-end-threaded knot-like topology that was found for SARS-CoV-2, to see if they all share the same 3-stem architecture seen in beta-CoVs (even pseudoknots from alpha-CoVs), and more generally to get a sense of the range of structures that form in bat-CoV pseudoknots (how many stems/loops, how they are arranged, etc…). We contend that qualitative comparisons are sufficient to answer these questions. A large part of the motivation for focusing on qualitative analysis is the variability that has been found in experimental structures of the SARS-CoV-2 pseudoknot—they agree on qualitative features (*e.g*., 5′-end threading, number and general arrangement of helices), but disagree on details (including tertiary contacts and some aspects of base-pairing). Trying to quantify details in computational models where experiments cannot agree does not seem to us to be an effective use of resources.

Nevertheless, we appreciate the reviewer’s desire to see additional replicates to address concerns about the reliability and reproducibility of the results, even with respect to the qualitative features of the models. We have therefore added numerous simulations (all 1 μs long) to verify the results and enhance confidence in the conclusions, focusing on threaded conformers because they are likely the most relevant biologically (as discussed above):

- We performed 2 more replicates of the threaded conformer of KU182958 (GLGC2) and LC469308 (Vs-CoV-1) without Mg^2+^, starting from the same initial coordinates as in the original manuscript, to show that we got effectively the same results, as discussed above in response to point 2 from reviewer 3.
- We extended our study to include MD simulations of the top 3 lowest-energy 3D structures predicted by FARFAR2 instead of only the lowest energy structure (again for threaded conformers without Mg^2+^), to verify that the same structural features were seen (qualitatively) and that the choice of 3D prediction did not bias the results, as discussed above in response to point 5 of reviewer 2 and point 3 of reviewer 3.
- We repeated simulations of the KU182958 and KF294276 threaded conformers in the presence of Mg^2+^ but placing the ions at different initial positions, to test if the choice of ion positions biased the result. As discussed above in response to reviewers 1 and 2, the qualitative structural features remained the same, but features like fluctuations and ion positions differed, suggesting that our simulations are not sufficient for such analyses, prompting us to remove comparisons of the effects of Mg^2+^ on fluctuations from the manuscript.

These additional simulations have confirmed the primary, qualitative conclusions of the manuscript, suggesting that they are reliable and robust, while identifying some minor parts of the analysis (namely, Mg^2+^ effects on fluctuations) as insufficiently reliable and therefore in need of removal.

We have revised the manuscript (abstract; Introduction, p. 4; Results, p. 11, 13) to make it clear that our focus is on qualitative features of the structure, and to add in the replication results (Fig. S5–S14).

*Minor, Required Revisions: [numbering added for convenience]*

*[3.] It is unclear how much sequence was used to generate the phylogenetic clusters in Figure 1. Was it the full length genomes or a region at the frameshifting site? And, if it is a region, how much was used? What alignment tool was used for the alignment?*

As noted above in response to point 1 of reviewer 2, the alignment and clustering were published previously (Ref. 17). To avoid the perception that we were “double publishing” the work on the alignment, we included only brief details and a condensed summary of the results in our original submission (Fig. 1), referring readers to Ref. 17 for more detail. However, given that we have now redone the alignment in response to comment 1 from reviewer 4, with many more sequences and a slightly different method, we have revised the manuscript (Methods, p. 5 Results, p. 7–9) to provide the full details of the alignment and clustering. Please see the discussion in response to comment 1 above (p. 14–15) for details on the alignment.

*[4.] For the secondary structure predictions, it is unclear what is meant by the “lowest-energy consensus pseudoknotted structure”. Does this mean that HotKnots and pKiss predicted the same structure in most cases or does it mean that the lowest energy of the two predictions was chosen?*

We thank the reviewer for pointing out the lack of clarity in our description of the 2D structure prediction. In the revised manuscript, we have added two more prediction algorithms in response to reviewer comments, hence the “consensus pseudoknotted structure” now means the pseudoknot that is predicted by the largest number of algorithms. Please note that in a few cases, only one algorithm predicted a pseudoknot structure, in which case this prediction is taken as the consensus. We have revised the text to clarify this point (Methods, p. 5, and Results p. 9), and also added Table S1 indicating which algorithms were part of the consensus.

*[5.] For the simulations performed with magnesium, how were the six ions placed? I am concerned that 1 us simulations are inadequate for equilibration of magnesium ions. This is discussed, for example, in [JCTC. 2016. 12: 3370] and references therein.*

Please see the response to point 3 from reviewer 2 on p. 5 above. To test the variability of the results when changing the placement of the ions, we repeated the MD simulations of the GLGC2 (KU182958) and Anlong-172 threaded conformers with different ion starting positions (3 replicates). The results suggest that 1 μs is indeed not enough to equilibrate the Mg^2+^ positions, as the reviewer worried: while the backbone structures in the ordered parts of the RNA were comparable for the different replicates, the ion positions were different.

However, we respectfully contend that reaching equilibrium is not needed for the purposes of our work: the primary aim of the simulations with Mg^2+^, as noted above, is to determine if Mg^2+^ induced any gross structural changes (such as disruptions of secondary structure or rearrangements of helix stacking/packing)—which we did not observe, for any of the pseudoknots we studied. Indeed, given the uncertainty about how many ions are present for each pseudoknot and where they are located (questions that have yet to be answered reliably for the SARS-CoV-2 pseudoknot, too), we contend that it is not meaningful to try to answer anything more detailed than such qualitative questions.

Nevertheless, the variability in these simulations suggests that our analysis of fluctuations in the presence of Mg^2+^ is not very reliable, given that the variation in fluctuations in L3 (for example) between simulations with different ion positions is similar to the differences in the fluctuations with and without Mg^2+^.

We have revised the manuscript to state more clearly the qualitative nature and purpose of the simulations with Mg^2+^, to include the analysis of replicates with Mg^2+^ in different initial positions and clarify the limits of what can be learned from the simulations, and to remove any discussion points in the results that imply or assume that these simulations are equilibrated.

*[6.] For the selection of the FARFAR models to use for simulations, how were the models for subsequent analysis chosen from the set of alternative structures?*

Reviewers 2 and 3 raised the same concern (point 5 from reviewer 2 on p. 6 above, point 3 from reviewer 3 on p. 12–13 above). Please see our responses on the pages indicated.

*[7.] A number of statements are qualitative, but should be made quantitative. For example, differences between models were stated to be “relatively minor.” KU182958 was stated to be “quite similar” to SARS-CoV-2. Fluctuations of L3 are stated to be “quite low” (immediately following the Fig. 4 caption).*

We thank the reviewer for noting instances where our descriptions were overly vague. We have revised the text to remove such statements (such as the first and third examples listed by the reviewer) or to amplify the qualitative descriptions with more detail. However, we respectfully feel that the statement about KU182958 being “quite similar” to SARS-CoV-2 is appropriate as it stands and does not need revision, given that we immediately expand on this statement to explain the ways in which they are quite similar (namely, “featuring a three-stem architecture where two of the stems interact as in a classic H-type pseudoknot and the third forms as an extended helix in what would otherwise be a large loop”).

More generally, we note that the use of qualitative statements is particularly appropriate in the context of this work, whose focus is ***not*** on detailed, quantitative descriptions of the structures, but rather on a qualitative survey of the range of structural features seen in bat-CoV pseudoknots and their similarities or differences, motivated by the reasons outlined above (p. 10, p. 13–14). Given that computational models in the absence of experiment can only ever be treated as approximations, that high-resolution experimental data or lower-resolution data in concert with simulations (as in Ref. 50 of the revised manuscript) are essential to get reliable information about the details of the structure, and that even in experimentally derived models there can be notable differences in the details, it does not seem to us to be fruitful to focus too much on comparing the minute details of the computational models (*e.g*., specific types of bond interactions at specific locations, or quantitative differences in fluctuations).

*[8.]* *It would be helpful if Figure 2 defined the colors in relationship to S1, S2, and S3. The reader needs to consult Fig. S1 to find the definitions.*

We thank the reviewer for noticing this oversight. We have revised the figure caption to define the colors more clearly, as suggested.

*[9.] The results of the clustering of conformations from the simulations was not detailed. For each set of coordinates, how many clusters were identified? Importantly, do clusters appear at multiple timepoints across trajectories or do clusters tend to appear once? If clusters appear transiently and do not recur, I am concerned that the simulations are unconverged. Here, multiple trajectories would also be helpful for testing convergence; for converged simulations I would expect cluster members to appear in independent trajectories when the trajectories are clustered together.*

The clusters are not transient, but recur at different points during the part of the simulation that is considered to be equilibrated, providing additional evidence that the simulations have converged. An example from the first beta-CoV we studied (GLGC2) is shown in Fig. R8 below, where the trajectories clearly shows recurring instances of the clusters. We identified the top 3 clusters, which together accounted for 70–100% of the frames in the equilibrated region for every trajectory. The new results showing convergence of the structures for the top clusters (outside of loops and flexible stems) obtained from replicates starting from the same initial structure or from other initial structures also support the notion that the simulations are equilibrated (at least in the absence of Mg^2+^).

We have revised the manuscript to describe the clustering results in more detail, adding panels showing the occupancy of the top 3 clusters in each of the simulations (Figs. S5, S8–S14).

**Fig. R8**: High occupancy and recurrence of top clusters in MD trajectories of GLGC2 pseudoknot threaded conformer (A) without and (B) with Mg^2+^, and unthreaded conformer (A) without and (B) with Mg^2+^. Only the last 500 ns are analyzed. Portions of the trajectory in which the top three clusters are occupied are indicated in color (cyan: cluster 1, orange: cluster 2, green: cluster 3).

*[10.] Much of the text compares fluctuations across simulations (across models or between simulations with and without magnesium). With single simulations for each model and solvent, it is unknown whether these differences are significant.*

After showing that the results with Mg^2+^ are dependent in part on details of the ion placement, we agree that comparisons of fluctuations in the simulations using Mg^2+^ are not reliable, and we have removed them (see p. 3 above). With regards to the simulations without Mg^2+^, adding replicates for the threaded structures as we have done now provides a sense of the variability in the fluctuations, allowing more reliable comparisons. We have added plots showing the average RMSF with standard error on the mean (as a quantitative measure of the variability) from these replicates to the revised manuscript, in Figs. S5, S9–S14. Nevertheless, given that the discussion of fluctuations is not a major part of the results, we have also restricted the discussion of the fluctuations in the revised manuscript to comparisons within a given model, not between models.

*[11.] The short stem-loop in a subset of KF294282 models was stated to dissociate. It would be helpful to know the timeframe of dissociation.*

The third stem took ~ 400–500 ns to dissociate in the 3-stem model discussed in the revised manuscript. We have revised the manuscript (Results, p. 18) to describe how long it took for S3 to dissociate.

**Most significant changes to manuscript**

- Abstract; Introduction, p. 4: Updated to mention new work on additional pseudoknots and emphasize qualitative focus of work, in response to all reviewers.
- Methods, p. 5; Results, p. 7–9; Fig. 1; Table S1, Fig. S1, File S1: Explained alignment and clustering procedures including new methods and validation added in response to reviews, updating Fig. 1 and adding supporting information, in response to reviewers 2 and 4.
- Methods, p. 5–6; Results, p. 9–10: Expanded and clarified methods for 2D and 3D structure prediction as well as motivation for strategy used, in response to reviewers 2 to 4.
- Methods, p. 7; Results p. 13; Fig. S6: explained how we verified trajectories were equilibrated and added replicates starting from the same initial structure, in response to reviewers 2 to 4.
- Results and Discussion, throughout: Relabelled all bat CoVs, in response to reviewer 2.
- Results and Discussion, throughout: Added results for 4 new alpha-CoV clusters found from new alignment on expanded set of sequences, in response to reviewer 4.
- Results, throughout: Removed discussion of fluctuations in simulations with Mg^2+^ and comparisons between pseudoknots, in response to reviewers 1, 2, and 4.
- Results, throughout: Added discussion of changes in base-pairing from 2D prediction to MD simulation, in response to reviewer 2.
- Results, p. 10–11 ff; Fig. S5–S14: Added replicate simulations to address concerns about reliability (replicates starting from each of the next-two-lowest energy 3D predictions for all pseudoknots with alternative predictions, replicates starting from the same initial structure for selected pseudoknots, and replicates starting with Mg^2+^ ions in different positions for selected pseudoknots), in response to all reviewers.
- Discussion, p. 21: Clarified the discussion of the structural diversity of alpha- vs beta-CoV pseudoknots, in response to reviewer 1.
- Discussion, p. 21: Clarified which parts of the pseudoknot sequences/structures varied the most, in response to reviewer 1.
- Discussion, p. 21–22: Clarified −1 PRF levels in different bat CoVs, in response to reviewer 2.
- Discussion, p. 22; Fig. S15: Added analysis of S3 flexibility, in response to reviewer 2.
- Fig. S4: Added figure showing SARS-CoV-2 structural models, in response to reviewers 2 and 3.
- Fig. S5, S8–S14: Added analysis of cluster occupancies within trajectories, in response to reviewer 4.
